# Transcription factor NF-κB is modulated by symbiotic status in a sea anemone model of cnidarian bleaching

**DOI:** 10.1038/s41598-017-16168-w

**Published:** 2017-11-22

**Authors:** Katelyn M. Mansfield, Nicole M. Carter, Linda Nguyen, Phillip A. Cleves, Anar Alshanbayeva, Leah M. Williams, Camerron Crowder, Ashley R. Penvose, John R. Finnerty, Virginia M. Weis, Trevor W. Siggers, Thomas D. Gilmore

**Affiliations:** 1 Department of Biology, Boston University, Boston, Massachusetts, 02215 USA; 2 Department of Genetics, Stanford University, School of Medicine, Stanford, California, 94305 USA; 3 Department of Integrative Biology, Oregon State University, Corvallis, Oregon, 97331 USA

## Abstract

Transcription factor NF-κB plays a central role in immunity from fruit flies to humans, and NF-κB activity is altered in many human diseases. To investigate a role for NF-κB in immunity and disease on a broader evolutionary scale we have characterized NF-κB in a sea anemone (*Exaiptasia pallida*; called Aiptasia herein) model for cnidarian symbiosis and dysbiosis (i.e., “bleaching”). We show that the DNA-binding site specificity of Aiptasia NF-κB is similar to NF-κB proteins from a broad expanse of organisms. Analyses of NF-κB and IκB kinase proteins from *Aiptasia* suggest that non-canonical NF-κB processing is an evolutionarily ancient pathway, which can be reconstituted in human cells. In Aiptasia, NF-κB protein levels, DNA-binding activity, and tissue expression increase when loss of the algal symbiont *Symbiodinium* is induced by heat or chemical treatment. Kinetic analysis of NF-κB levels following loss of symbiosis show that NF-κB levels increase only after *Symbiodinium* is cleared. Moreover, introduction of *Symbiodinium* into naïve Aiptasia larvae results in a decrease in NF-κB expression. Our results suggest that *Symbiodinium* suppresses NF-κB in order to enable establishment of symbiosis in Aiptasia. These results are the first to demonstrate a link between changes in the conserved immune regulatory protein NF-κB and cnidarian symbiotic status.

## Introduction

Many members of the phylum Cnidaria engage in mutualistic endosymbioses with photosynthetic dinoflagellates. In the case of reef-building corals, these symbiotic relationships are essential for the trophic and structural foundation of coral reef ecosystems^1^. Cnidarian bleaching is a dramatic example of dysbiosis that occurs when these dinoflagellate symbionts exit or are expelled by host tissues^1^. It is now clear that coral bleaching, which often leads to coral mortality and collapse of the associated ecosystem^2^, is caused by environmental perturbations, especially elevated water temperature associated with global warming^3^. Analyses of changes in whole animal mRNA expression patterns have suggested that the cnidarian host innate immune system is involved in symbiosis, but such reports have been conflicting. In some cases, innate immunity has been implicated in tolerating and/or promoting the survival of the beneficial photosynthetic algae in their cnidarian hosts^4–8^, whereas other studies have suggested that innate immunity is involved in symbiosis dysfunction or dysbiosis^9,10^. Moreover, such RNA-Seq studies are limited in that they do not characterize changes in the activity or cell-type expression of cellular proteins that play roles in the healthy establishment of symbiosis or in dysbiosis and bleaching.

The NF-κB transcription factor family has been widely studied for its roles in a variety of immune-related processes, as well as diseases, in animals ranging from insects to mammals^11^. Based primarily on genomic and transcriptomic sequencing, it is clear that NF-κB-like transcription factors are present in some of the earliest emerging animal phyla (e.g., Porifera, Cnidaria) and some closely related outgroups to Metazoa (e.g., Filasterea)^11,12^. However, little is known about the activity, regulation, or biological roles of NF-κB in these more basal eukaryotes^11,12^.

All NF-κB proteins have an N-terminal DNA-binding/dimerization domain called the Rel Homology Domain (RHD)^13^. In mammals, the NF-κB superfamily comprises five related transcription factors that fall into two subgroups: the NF-κB proteins (p52/p100; p50/p105) and the Rel proteins (c-Rel, p65/RelA, RelB). By comparison of protein sequences^14^ and DNA-binding site preferences^15^, the RHDs of human NF-κB proteins are more similar to one another than they are to the Rel proteins. *Drosophila* has a single NF-κB-like protein (Relish) and two Rel-like proteins (Dorsal, Dif). Sponges, cnidarians, and *Capsaspora owczarzaki* ^16^, a unicellular holozoan, have single NF-κB-like proteins that are more similar by amino acid sequence comparison to vertebrate NF-κB proteins than to Rel proteins^14,17^.

There are also differences between the NF-κB and Rel subclasses in sequences outside of the RHD^13^. The NF-κB proteins have a C-terminal inhibitory domain, which consists of a series of ankyrin (ANK) repeats that must be removed by proteolysis to activate the DNA-binding activity of the transcription factor. For example, vertebrate p100 and p105 proteins are processed by the proteasome to their active forms, p52 and p50, respectively. In contrast, Rel-like proteins do not have ANK repeats, do not require processing for activation, and contain C-terminal transactivation domains.

In vertebrates, Rel/NF-κB dimers are generally held in the cytoplasm in an inactive state by interaction with NF-κB inhibitor proteins called IκBs. In mammals, there are two distinct NF-κB activation pathways: the canonical and the non-canonical pathways^18^. In the canonical pathway, a dimer such as p50/65 is bound to a separate IκB inhibitor, and this complex is activated to enter the nucleus when the IκB protein is phosphorylated by an IκB kinase (IKKβ), which promotes IκB ubiquitination and proteasomal degradation^18^. In the non-canonical pathway, the RelB/p100 dimer is activated by IKKα-dependent phosphorylation of p100 at a cluster of serine residues C-terminal to the ANK repeats^19^. This phosphorylation event marks p100 for ubiquitination and proteasome-mediated proteolysis of the C-terminal ANK domain, allowing the p52/RelB dimer to enter the nucleus^19^. In *Drosophila*, the single NF-κB protein (Relish) is activated by caspase-mediated proteolytic cleavage at a specific site C-terminal to the RHD, which then removes the C-terminal ANK repeats^20^. Although activation of NF-κB is generally considered to be an induced process, in some normal resting cell types, such as mammalian B cells, NF-κB proteins are constitutively found in the nucleus^21^.

We have had a continuing interest in characterizing the function and regulation of NF-κB in early branching animal phyla, focusing to date on cnidarians such as the sea anemone *Nematostella vectensis* ^22,23^. Even though NF-κB proteins from most early animal phyla have an overall structure similar to the mammalian RHD-ANK repeat bipartite configuration, there is considerable variation among cnidarians^23^. For example, the single NF-κB-like protein in *Nematostella* contains an intact RHD, but lacks the C-terminal ANK repeat domain. Instead, *Nematostella* has an IκB-like ANK repeat domain protein encoded by a separate, unlinked gene^23^, but the Nv-IκB is, by certain phylogenetic criteria, most similar to the C-terminal sequences of mammalian NF-κB proteins^24^. However, the Nv-IκB protein has no discernable C-terminal sites of IKK phosphorylation (like NF-κB p100)^22^.

Due to reports of a correlation between immune gene expression and bleaching in certain corals^9,25^, as well as the pervasive role of NF-κB in immunity and disease across a broad evolutionary expanse^22,23,24^, we have now characterized NF-κB signaling in the sea anemone *Exaiptasia pallida*, commonly called Aiptasia, with the goal of gaining an understanding of molecular pathways involved in cnidarian dysbiosis. Aiptasia has served as a convenient model for cnidarian symbiosis because it engages in heat-sensitive symbiosis with the dinoflagellate *Symbiodinium* spp., the same genus of symbionts required for viability in all reef-building stony corals. In this report, we demonstrate increases in NF-κB protein expression, activity, and tissue distribution in Aiptasia following loss of symbiosis. Overall, these results suggest that changes in an NF-κB-dependent pathway are involved in the establishment of symbiosis in Aiptasia, and these results have implications for other cnidarian host-symbiont interactions.

## Results

### The Aiptasia NF-κB protein has similarities in structure, activity, and regulation to mammalian non-canonical NF-κB

**Figure 1:**
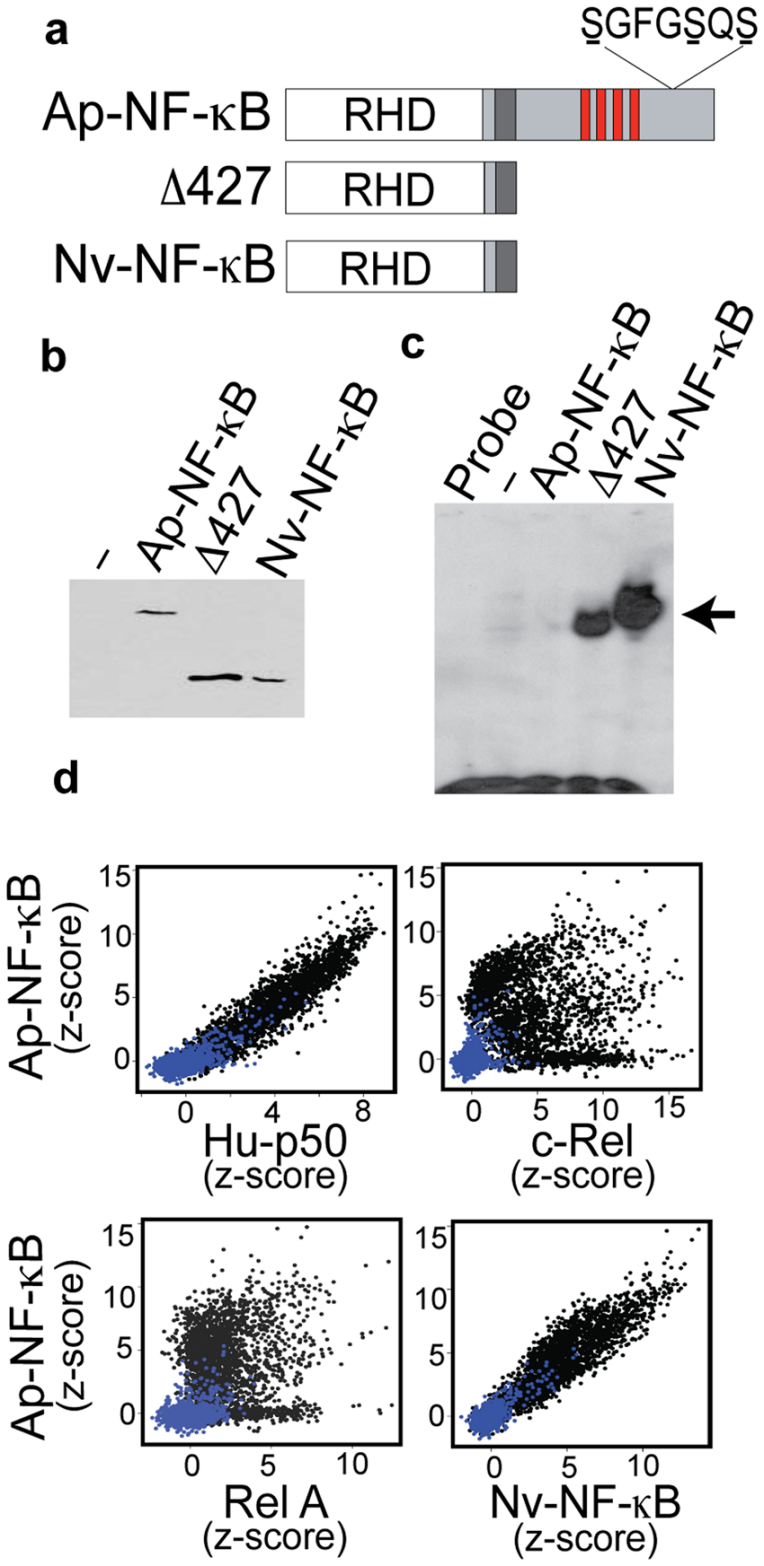
Conserved DNA-binding activity of Ap-NF-κB. (**a**) Shown are the generalized structures of Ap-NF-κB, a C-terminal truncation mutant of Ap-NF-κB (Δ427), and the naturally shortened Nv-NF-κB protein. White: Rel homology domain (RHD). Dark grey: Glycine rich region. Red: Ankyrin repeats. Ap-NF-κB putative IKK-phosphorylation sites shown. (**b**) Western blot of extracts from human 293 cells transfected with FLAG-tagged versions of the indicated proteins. The relevant section of the scanned film is shown; no other bands were detected on the gel. (**c**) An NF-κB-site EMSA of extracts from 293 cells transfected with the indicated proteins. The arrow indicates the position of the DNA-protein complex. The free probe is seen as a black band across the bottom of the image. (**d**) Comparison of the PBM-based DNA-binding profile of Aiptasia NF-κB to human p50, c-Rel, Rel-A, and *Nematostella vectensis* NF-κB (Nv) to 2592 κB sites (black dots) and 1195 random background sites (blue dots).

To investigate whether NF-κB plays a role in the regulation of cnidarian symbiosis, we chose to analyze NF-κB signaling using the model sea anemone Aiptasia. The analysis of transcriptomic and genomic databases of Aiptasia revealed a single gene encoding an NF-κB-related protein. The *Aiptasia* NF-κB protein (Ap-NF-κB) has a structural organization that is analogous to mammalian NF-κB p100: an N-terminal RHD, followed by a glycine-rich region (GRR), a series of ANK repeats, and putative regulatory sites of serine phosphorylation by IKK (Fig. 1a and Supplementary Fig. 1).

To analyze the biological regulation and molecular properties of Ap-NF-κB, we first created pcDNA-FLAG mammalian expression vectors for full-length Ap-NF-κB and a C-terminally truncated mutant (Δ427) that approximates the structure of a processed form of Ap-NF-κB. These two vectors expressed appropriately sized proteins in human 293 cells (Fig. 1b). The Ap-NF-κBΔ427 protein migrated similarly to the *Nematostella* NF-κB protein (Nv-NF-κB), which lacks a C-terminal IκB-like domain^22–24^. In an electrophoretic mobility shift assay (EMSA) using an NF-κB-site probe, extracts from 293 cells transfected with the Δ427 mutant, but not full-length Ap-NF-κB, showed a high level of DNA-binding activity, which was comparable to that seen with Nv-NF-κB (Fig. 1c)^22^.

To determine the overall DNA binding site specificity of Ap-NF-κB, we assessed the DNA-binding profile of bacterially expressed, affinity purified Ap-NF-κB in a protein-binding microarray (PBM) consisting of 2592 κB-type sites and 1195 random background sequences (Supplementary Data 1). We have previously used similar PBM analyses to compare the DNA binding site preferences of mammalian NF-κB family proteins^15^. By comparing z-scores for binding to specific sites on the PBM, we found that the DNA-binding profile of Ap-NF-κB is similar to human NF-κB p50 and Nv-NF-κB, but is distinct from human c-Rel and RelA (Fig. 1d). Furthermore, a comparison of the DNA-binding profile of a variety of other Rel/NF-κB proteins across a broad evolutionary expanse clearly places Ap-NF-κB among the subfamily of NF-κB proteins (Supplementary Fig. 2). In addition, a comparison of the general structure of the Ap-NF-κB protein shows that it is most similar to NF-κB subfamily proteins (Supplementary Fig. 3).

**Figure 2:**
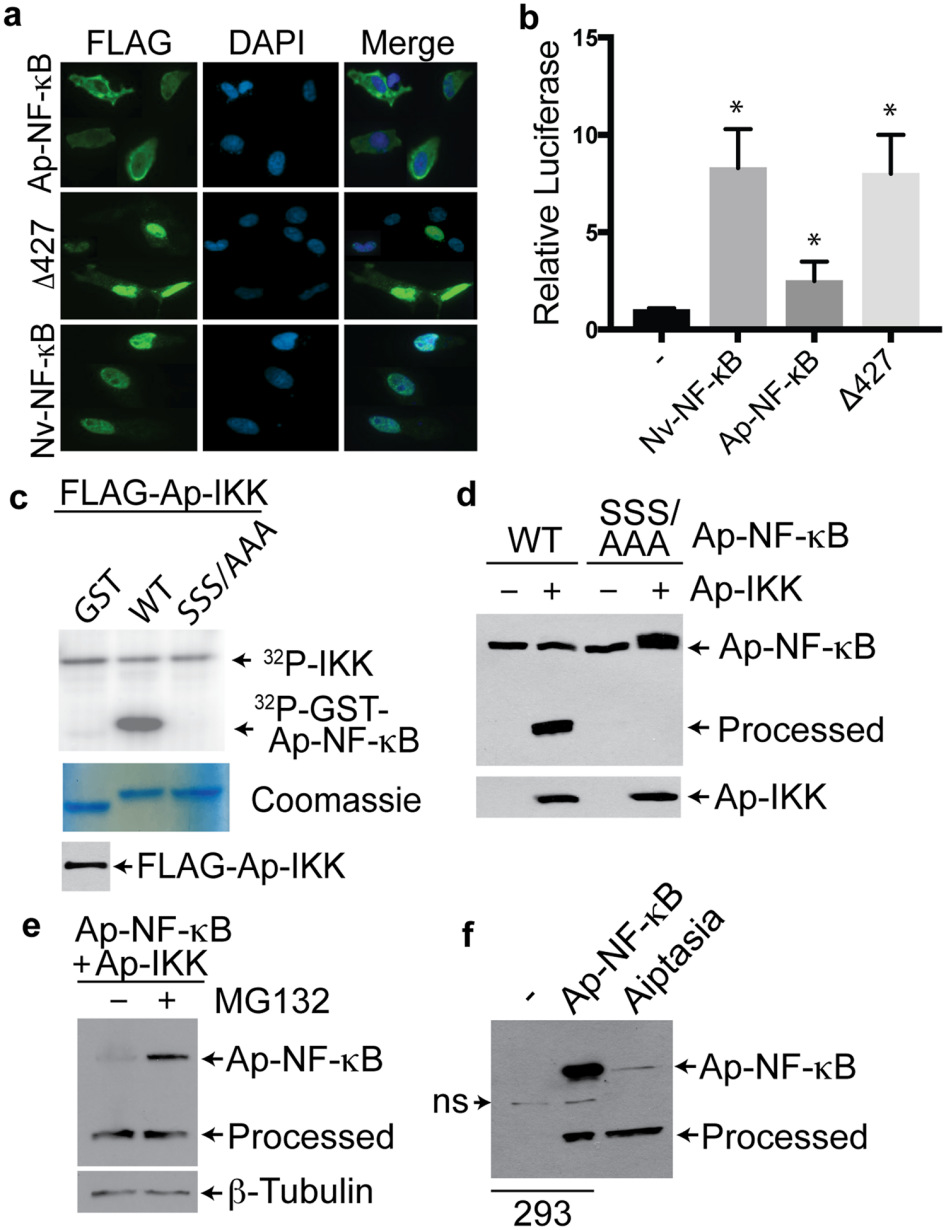
Processing of Ap-NF-κB. (**a**) Indirect immunofluorescence (green) using anti-FLAG primary antiserum of DF-1 chicken fibroblasts transfected with the indicated FLAG-tagged proteins. Nuclear staining with DAPI is shown in blue. (**b**) An NF-κB-site luciferase reporter gene assay was performed in 293 cells with the indicated proteins. Luciferase activity is relative to that seen with the vector control (1.0), and is the average of three experiments done in triplicate. Standard error is indicated. (**c**) An *in vitro* kinase assay using FLAG-Ap-IKK and GST-fusion proteins containing C-terminal sequences (amino acids 783–812) of wild-type and 3X Ser-Ala mutant Ap-NF-κB. The top image is a phosphorimage of ^32^P-labeled proteins, showing the relevant portion of the image; the free ATP images at the bottom on the gel are not shown. The bottom image shows a Coomassie blue-stained gel of GST proteins used in the kinase assay. An anti-FLAG blot of the 293 cell extract was also performed to confirm Ap-IKK expression (not shown). (**d**) 293 cells were transfected with full-length wild-type or 3X Ser-Ala mutant Ap-NF-κB in the absence (−) or presence (+) of an Ap-IKK expression vector. Extracts were analyzed by anti-Ap-NF-κB Western blotting, and the full-length and processed forms of Ap-NF-κB are indicated. At the bottom is an anti-FLAG Western blot showing expression of Ap-IKK, as determined by its migration against molecular weight standards. (**e**) 293 cells were transfected with full-length Ap-NF-κB and Ap-IKK in the absence (−) or presence (+) of proteasome inhibitor MG132. Extracts were analyzed by anti-Ap-NF-κB Western blotting. The filter was then stripped and probed for β-tubulin as a loading control. (**f**) Anti-Ap-NF-κB Western blotting of 293 cells expressing full-length and processed forms of Ap-NF-κB and lysates from symbiotic Aiptasia anemones. The only bands seen on the film are shown. ns, non-specific band.

NF-κB p100 protein activation requires phosphorylation by an upstream IKK, which is followed by C-terminal proteolytic processing and nuclear localization of the mature NF-κB p52 protein. Therefore, we analyzed Ap-NF-κB for these central properties of NF-κB signaling and regulation. First, by immunofluorescent staining of transfected chicken fibroblasts, we found that the full-length Ap-NF-κB localized exclusively to the cytoplasm, whereas the C-terminally truncated Ap-NF-κBΔ427 mutant was exclusively in the nucleus (Fig. 2a). As a control, we show that Nv-NF-κB, which has a structure similar to the Δ427 mutant, was also exclusively in the nucleus of transfected cells (Fig. 2a), consistent with our previous results^22^. Secondly, we found that Ap-NF-κBΔ427 transactivated a multimeric NF-κB-site reporter in 293 cells to approximately the same extent as Nv-NF-κB (Fig. 2b)^22^. In contrast, full-length Ap-NF-κB showed little ability to activate the reporter above vector control levels (Fig. 2b). Thus, our results show that the C-terminally truncated Ap-NF-κBΔ427 protein has increased DNA-binding activity, nuclear localization, and transactivation ability as compared to full-length Ap-NF-κB. Ap-NF-κBΔ427 shares these three properties with the processed p52 form of the mammalian NF-κB p100 protein, suggesting that the removal of Ap-NF-κB’s C-terminal ANK repeat is necessary for activation of the protein.

In the mammalian non-canonical pathway, activated IKKα initiates processing of NF-κB p100 by phosphorylating three serine residues downstream of the p100 C-terminal ANK repeats, which then promotes processing of p100 to p52 by the proteasome^19^. Ap-NF-κB contains three serine residues in a similar arrangement and location as the serine residues in human p100 that are phosphorylated by IKKα (Supplementary Fig. 1). A BLAST analysis identified a single IKK-like protein in Aiptasia, which, by MEME and phylogenetic analyses, was indicated to be an IKKα/β-like protein homolog (Supplementary Fig. 4 and Supplementary Data 2–4). In an *in vitro* kinase assay, Aiptasia IKK (Supplementary Data 5) and human IKKα and β can all phosphorylate a peptide containing C-terminal sequences (aa 783–812) of Ap-NF-κB, but cannot phosphorylate the same peptide in which the three Ser residues are converted to Ala (Fig. 2c and Supplementary Fig. 5). Moreover, co-transfection of 293 cells with an expression vector for Ap-IKK induces processing of wild-type Ap-NF-κB to a protein of approximately 52 kDa, but Ap-IKK did not induce processing of the SSS/AAA mutant of Ap-NF-κB under the same conditions (Fig. 2d). Ap-IKK-induced processing of Ap-NF-κB in 293 cells is reduced in the presence of the proteasome inhibitor MG132 (Fig. 2e). Co-transfection of Ap-NF-κB with human NIK (to activate endogenous IKKα) or a constitutively active human IKKβ also induced processing of Ap-NF-κB in 293 cells (Supplementary Fig. 6). Finally, we show that lysates from Aiptasia contain forms of Ap-NF-κB that co-migrate with both full-length and processed forms of Ap-NF-κB that were detected upon expression in 293 cells (Fig. 2f). These results identify phosphorylation sites and conditions that are necessary for Ap-IKK induced processing of Ap-NF-κB, and suggest that similar processing events occur in Aiptasia. In contrast, NIK, IKKβ, and Ap-IKK did not induce cleavage of the sponge Aq-NF-κB protein, which lacks clear IKK phosphorylation sites (Supplementary Fig. 3), upon co-transfection in 293 cells (Supplementary Fig. 7).

Taken together, these results show that Ap-NF-κB has structural, functional and regulatory similarities to the non-canonical mammalian NF-κB protein p100, and that these properties are conserved even upon expression of Ap-NF-κB in human cells. Furthermore, the co-migration of full-length and processed forms of Ap-NF-κB expressed in 293 cells with two forms of Ap-NF-κB detected in extracts from anemones suggests that a similar processing event occurs in Aiptasia.

### NF-κB mRNA, protein, and activity increase with loss of symbiosis in Aiptasia

**Figure 3:**
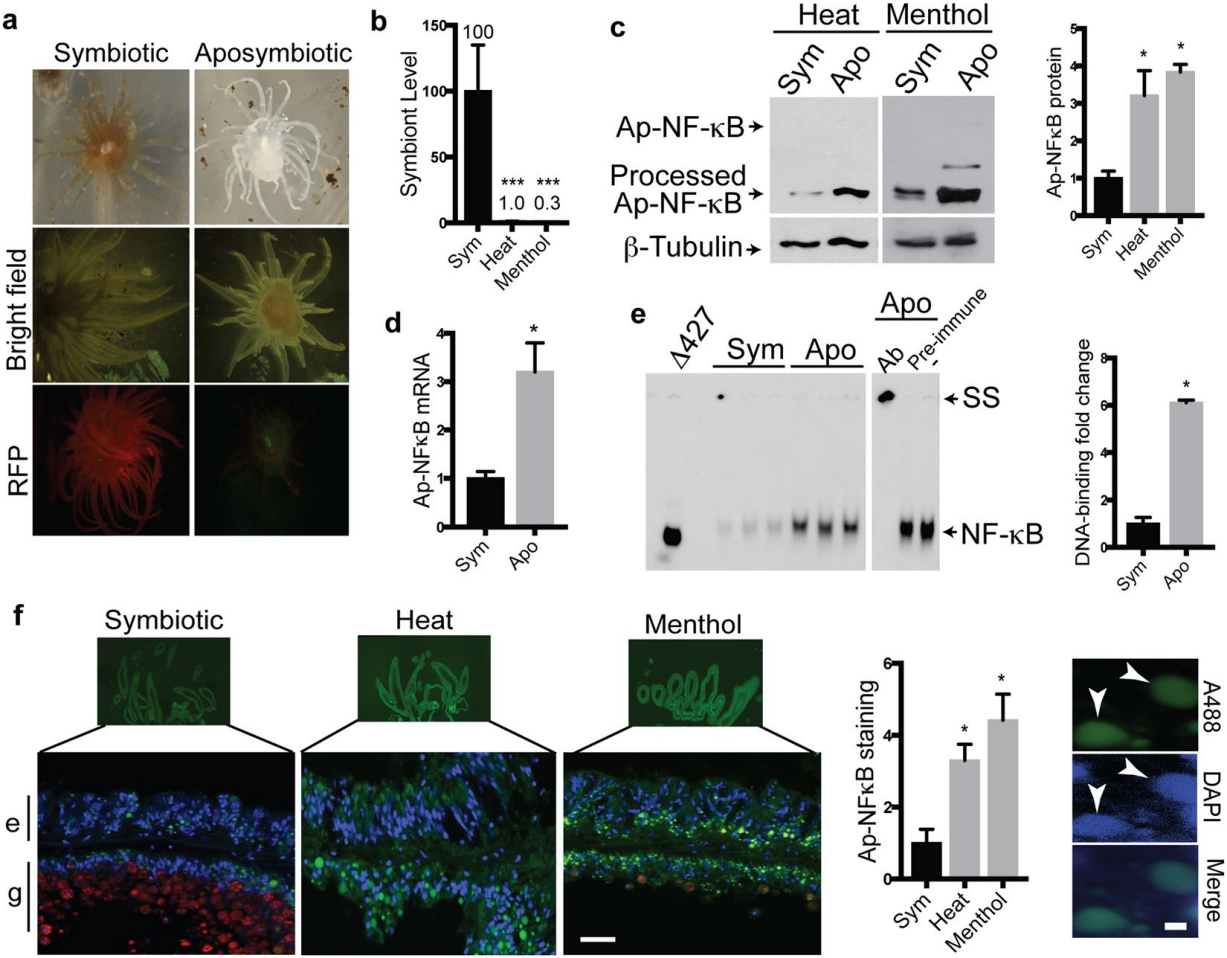
Increased levels and activity of Ap-NF-κB in bleached anemones. (**a**) Aiptasia were maintained in the symbiotic state or rendered aposymbiotic by treatment with menthol, and then imaged by light microscopy (top) or fluorescence microscopy. In the bottom image, *Symbiodinium* fluoresce red due to chlorophyll autofluorescence. (**b**) qPCR using primers against *Symbiodinium* Clade B 28 S rRNA was performed on Aiptasia treated as indicated. *Symbiodinium* 28 S rRNA levels are relative to the amount in symbiotic control anemones (100). n = 3. Statistical significance compared to sym determined using an unpaired T-test: ***p ≤ 0.001 (**c**) Aiptasia extracts (symbiotic, heat-bleached or menthol-bleached) were analyzed by anti-Ap-NF-κB Western blotting. The relative amount of Ap-NF-κB protein was determined by band quantitation using ImageJ on Western blots of three biological replicates per group, and values were normalized to the amount of β-tubulin in each sample. Statistical significance of the increases in Ap-NF-κB in aposymbiotic animals as compared to those in symbiotic anemones was determined using an unpaired T-test: *p < 0.05. The Ap-NF-κB bands were detected as in Fig. 2. β-tubulin was detected following stripping of the primary anti-Ap-NF-κB Western blot. (**d**) Relative amounts of Ap-NF-κB mRNA were determined by qPCR of RNA from symbiotic and menthol-bleached *Aiptasia*. Values are relative to the amount of NF-κB mRNA in control symbiotic anemones (1.0). Sym n = 6, apo n = 3. Statistical significance compared to sym was determined using an unpaired T-test: *p < 0.05. (**e**) An NF-κB-site EMSA was performed using extracts from symbiotic (SYM) and menthol-bleached (APO) *Aiptasia*. Δ427 indicates that extracts from 293 cells transfected with pcDNA-FLAG-Ap-NF-κBΔ427 were used (as in Fig. 1c). Supershifts were performed with Ap-NF-κB antiserum (Ab), preimmune serum, or no antiserum (−). The relevant portion of the image containing the NF-κB-DNA complex (NF-κB) or the supershifted (SS) complex is shown; not shown is the free probe that migrates at the bottom on the gel (see Fig. 1c). (**f**) On the left is shown indirect immunofluorescence analysis of whole-body sections from symbiotic and aposymbiotic (heat- and menthol-threated) anemones. 20 μm frozen sections were stained with Ap-NF-κB primary antiserum and Alex Fluor 488-conjugated secondary antiserum. Ap-NF-κB staining (green); DAPI staining (blue); *Symbiodinium* cells (red, autofluorescence). Top images were taken at 4X, bottom images at 40X (scale bar: 20 μm). e, epidermis; g, gastrodermis. The graph shows quantitation using ImageJ of relative NF-κB puncta in three different sections of tentacles for each type of anemone. Statistical significance compared to Sym was determined using an unpaired T-test: *p < 0.05. The right panels are high magnification images showing that NF-κB staining (A488) coincides with nuclear DAPI staining in anemone sections. Scale bar: 2 μm (right).

To determine whether Ap-NF-κB is affected by the symbiotic status of Aiptasia, we induced loss of symbiosis in anemones by two commonly used methods, namely elevated water temperature and menthol treatment. Elevated temperature has been used to mimic climate change-induced bleaching, and in the laboratory, symbiotic anemones can be bleached by increasing water temperature from 25 °C to 32 °C for 6 days. Menthol treatment produces bleached anemones after just three days of treatment^26^. With either treatment, the anemones take on a strikingly bleached appearance (Fig. 3a, top right), and they show greatly reduced numbers of symbionts by fluorescence (Fig. 3a, bottom right) and by qPCR of symbiont 28S RNA (Fig. 3b). In this paper, we refer to our heat- and menthol-treated anemones as aposymbiotic based on the greatly reduced numbers of symbionts present after either treatment.

To determine whether NF-κB expression is altered by symbiotic status in Aiptasia, we compared the protein and mRNA levels of Ap-NF-κB between symbiotic and aposymbiotic animals. The levels of processed Ap-NF-κB protein were increased approximately three-fold in heat- and menthol-induced aposymbiotic anemones as compared to symbiotic anemones (Fig. 3c) and similarly, the level of Ap-NF-κB mRNA was increased by approximately three-fold in menthol-induced aposymbiotic anemones as compared to symbiotic anemones (Fig. 3d). There was also an approximately six-fold increase in NF-κB site DNA-binding activity in extracts from menthol- and heat-induced aposymbiotic Aiptasia (Fig. 3e; Supplementary Fig. 8). That the increased DNA-binding activity in aposymbiotic anemone extracts is due to Ap-NF-κB is suggested by the similar migration of the DNA-protein complex with the complex generated by the Δ427 protein expressed in 293 cells and by the ability of Ap-NF-κB antiserum (but not preimmune serum) to supershift the complex from aposymbiotic anemones (Fig. 3e; Supplementary Fig. 8).

To compare the whole organism pattern of Ap-NF-κB expression, we performed anti-Ap-NF-κB immunofluorescence on cryosections of symbiotic and heat- and menthol-induced aposymbiotic Aiptasia (Fig. 3f). At low magnification imaging of tentacle cross-sections (Fig. 3f, top), Ap-NF-κB staining was noticeably brighter in aposymbiotic anemones. At higher magnification (Fig. 3f, bottom), NF-κB was detected in symbiotic animals in a few cells primarily in the epidermis and away from cells containing intracellular symbionts located in the gastrodermis. After heat- or menthol-induced bleaching, there was an approximately three- to four-fold increase in NF-κB-positive cells, which are mostly in the gastrodermis where symbionts were greatly reduced in number. The three- to four-fold increase in Ap-NF-κB-positive cells seen in aposymbiotic Aiptasia tissue sections is consistent with the increased levels of Ap-NF-κB protein seen by Western blotting of whole animal extracts (Fig. 3c). The staining of Ap-NF-κB in tissue sections from both symbiotic and bleached anemones coincides with nuclear DAPI staining (Fig. 3f, right). Overall, these results show that loss of *Symbiodinium* from Aiptasia is correlated with increased NF-κB protein levels and DNA-binding activity as well as expanded tissue expression of nuclear NF-κB.

### Increased NF-κB protein expression in Aiptasia follows loss of symbiosis

**Figure 4:**
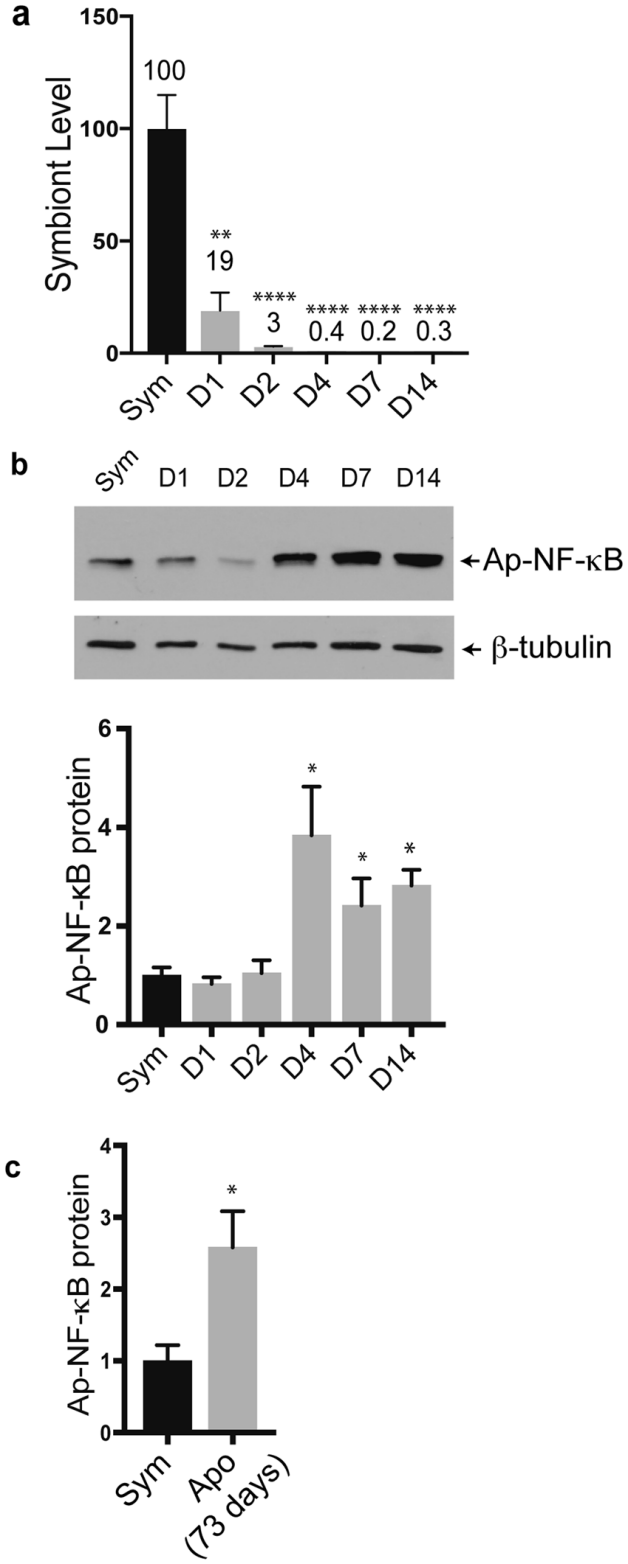
Increased expression of NF-κB protein following loss of symbiosis. (**a**) qPCR of *Symbiodinium* Clade B 28 S rRNA was performed on RNA from anemones collected at the indicated time-points during or after menthol treatment. Anemones were incubated with menthol on D1-D3 and were placed in fresh ASW from D4-D14. *Symbiodinium* 28 S rRNA levels are relative to the amount of 28 S rRNA in symbiotic animals (100). Sym n = 6, all other groups n = 3. Statistical significance for each group compared to symbiotic was determined using an unpaired T-test: **p < 0.01, ****p < 0.0001. (**b**) Representative anti-Ap-NF-κB Western blot of extracts from symbiotic and menthol-treated Aiptasia extracts prepared from animals treated as in (**a**). Shown is the portion of the gel containing the processed form of Ap-NF-κB. At this exposure, the much weaker full-length form of Ap-NF-κB was not seen (e.g., see Fig. 2f). The filter was then stripped and reprobed for β-tubulin. Quantitation (bottom) is based on the relative amount of Ap-NF-κB protein from band quantitation using ImageJ of all biological replicates for each group. In each case, Ap-NF-κB values normalized to the amount of β-tubulin in each sample, and values are relative to the amount of NF-κB in the Sym animals (1.0). Statistical significance was determined using an unpaired T-test for each group as compared to the symbiotic control group. *p < 0.05. (**c**) Quantitation of relative Ap-NF-κB protein from symbiotic anemones (1.0) compared to anemones that were maintained in the aposymbiotic for 73 days. NF-κB was quantified from Western blots as described for (**b**). Sym n = 5, Apo n = 3. Statistical significance was determined using an unpaired T-test as above: *p < 0.05.

To investigate the kinetics of increased NF-κB expression during loss of symbiosis, Ap-NF-κB expression and symbiont levels were quantified at multiple time-points during the process of menthol-induced loss of symbiosis. In these experiments, symbiotic anemones were treated for three days with menthol and were then given regular seawater for the remainder of the experiment. Aiptasia were collected during the first two menthol treatments (Days 1 and 2, i.e., D1 and D2) and at three time-points after menthol treatment when the anemones were in seawater (D4, D7 and D14). As shown by qPCR, menthol treatment induces a rapid and progressive loss of symbionts, with an ~80% loss of symbionts at D1 as compared to control anemones, and an approximately 99% decrease in symbionts by D4 (Fig. 4a). After D4, the symbiont densities remained at this low level through D14 (Fig. 4a), indicating that the symbionts were not repopulating the animal after removal of the menthol. By Western blotting, Ap-NF-κB protein levels in anemones at D1 and D2 of menthol treatment were similar to those seen in control, untreated animals (Fig. 4b). However, by D4 Ap-NF-κB protein levels were statistically increased (i.e., 3.8-fold) as compared to control anemones, and the levels of Ap-NF-κB protein remained statistically higher than in control animals even at D14 (Fig. 4b). In addition, anemones that have been kept aposymbiotic for a longer period of time, i.e. 73 days, have significantly higher NF-κB expression as compared to symbiotic anemones (Fig. 4c). These results show that increased levels of NF-κB occur only after there has been a substantial loss of symbionts and that the increase in NF-κB protein levels is maintained in aposymbiotic animals for over two months after bleaching is initially induced.

### Introduction of *Symbiodinium* into naïve Aiptasia larvae leads to reduced NF-κB expression

**Figure 5:**
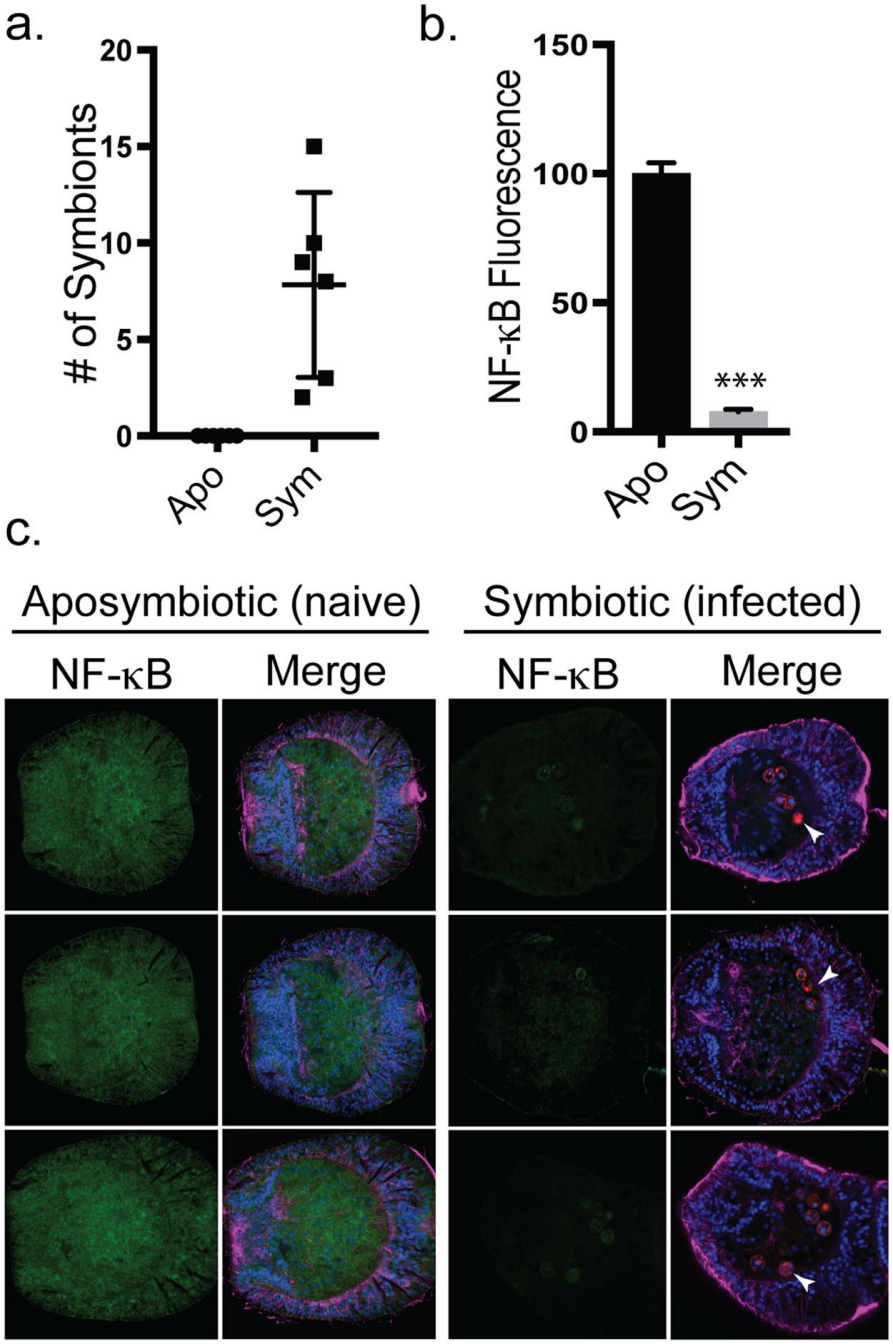
Infection with *Symbiodinium* reduces NF-κB expression in Aiptasia larvae. (**a**) NF-κB protein levels were measured by indirect immunofluorescence of aposymbiotic larvae and larvae infected with SSB01 *Symbiodinium*. The number of symbiont cells in individual larvae were counted by manually scanning through the z-plane in confocal images. Apo, n = 8; Sym, n = 6. No symbionts were seen in Apo larvae. (**b**) Ap-NF-κB fluorescence in Apo and Sym Aiptasia larvae was quantified using Corrected Total Cell Fluorescence (CTCF) of confocal images measured using ImageJ. Values are relative to the CTCF value of Apo larvae (100). Five larvae were quantified for each group. ***p < 0.0001. (**c**) Representative immunofluorescence images of naïve aposymbiotic larvae and larvae infected with *Symbiodinium*. NF-κB panels, NF-κB staining alone. Merged panels: Green, NF-κB; Blue, nuclei; Red, *Symbiodinium* (e.g., white arrows); Magenta, α-tubulin.

Given that loss of *Symbiodinium* was correlated with increased Ap-NF-κB expression, we hypothesized that introduction of *Symbiodinium* into aposymbiotic Aiptasia would decrease NF-κB expression. For these experiments, naturally and completely aposymbiotic Aiptasia larvae were inoculated with *Symbiodinium*, and Ap-NF-κB expression was then assessed using indirect immunofluorescence of whole-mount specimens. This approach was used for two reasons: 1) we could ensure that the larvae were aposymbiotic and had never seen *Symbiodinium*; and 2) the population of anemones with *Symbiodinium* could be done within a much shorter period (i.e., about 11 days) than the lengthy period (months) required for repopulating aposymbiotic adults. In these experiments, larvae at 4–5 days post-fertilization (dpf) were inoculated with *Symbiodinium*, and then 5–6 days after infection the larvae were fixed and stained with Ap-NF-κB antiserum. In larvae that were successfully infected, the numbers of symbionts present ranged from 2–15 cells (mean of 7.8 *Symbiodinium/*larva) (Fig. 5a). Larvae infected with *Symbiodinium* showed a 92% decrease in Corrected Total Cell Fluorescence (CTCF) for Ap-NF-κB as compared to control uninfected larvae (Fig. 5b,c). This result demonstrates that Ap-NF-κB protein expression is down-regulated in Aiptasia upon infection with S*ymbiodinium*.

## Discussion

Overall, the results presented herein correlate NF-κB transcription factor expression and activity with symbiotic status in Aiptasia, demonstrating that NF-κB expression is suppressed by the establishment of symbiosis and is increased by loss of symbiosis. The association between increased NF-κB expression/activity and loss of symbiosis in Aiptasia is especially noteworthy given that Aiptasia is a laboratory model for the pervasive climate change-induced coral bleaching that is occurring in the environment. Our results also suggest that a form of non-canonical NF-κB processing is present in the sea anemone Aiptasia. We show that basal NF-κB proteins have DNA-binding specificities and overall structures that are more similar to human NF-κB proteins than to human Rel proteins. Moreover, the Aiptasia NF-κB protein undergoes processing in human cells when human or Aiptasia IKK proteins are overexpressed.

Mammals and some arthropods have multiple Rel/NF-κB proteins, whereas all basal metazoan or pre-metazoans have single NF-κB-like proteins, based on genomic and phylogenetic studies^11,14^. Our PBM-based DNA-binding analyses also indicate that invertebrate NF-κB-like proteins (from *Capsaspora*, sponge, anemones, and *Drosophila*) are more similar to human NF-κB p50 and p52 proteins than to c-Rel and p65 proteins (Supplementary Fig. 2). Thus, it is almost certain that NF-κB proteins arose first and later expanded and diversified into the Rel family, consistent with earlier predictions^27^.

Our PBM analyses demonstrate that NF-κB DNA-binding specificity is highly conserved across a great evolutionary distance. That is, there is little difference between the DNA-binding site profiles of human NF-κB p50 and either sponge or anemone NF-κB proteins. These results suggest that there has not been substantial diversification in the DNA-binding site preference of NF-κB proteins along the main evolutionary branch from sponges to humans. Therefore, we propose that differences in gene networks required for new biological processes controlled by NF-κB proteins arose primarily due to mutations in the transcriptional regulatory DNA elements of new target genes. For example, the specialized developmental processes (cnidocyte formation and dorsal-ventral polarity) controlled by *Nematostella* and *Drosophila* NF-κB proteins^28,29^ likely came about due to the acquisition of NF-κB binding sites in DNA regulatory elements of genes required for cnidocyte development and embryonic polarity, respectively.

In the vertebrate non-canonical pathway, the inactive, cytoplasmic NF-κB complex is a p100-RelB heterodimer. The active p52/RelB heterodimer is generated by activation of the upstream kinase NIK that phosphorylates IKKα which then phosphorylates C-terminal residues of p100 to induce proteasomal processing of p100 to p52. As we show here, Ap-NF-κB has three C-terminal Ser residues that are similar to those in human p100 (Supplementary Fig. 1) and can be phosphorylated by human and Aiptasia IKKs (Fig. 2c and Supplementary Fig. 5). Moreover, overexpression of human NIK (and consequent activation of IKKα), human IKKβ, or Ap-IKK in human 293 cells can induce processing of Ap-NF-κB (Fig. 2d and Supplementary Fig. 6) to essentially the same extent as IKK phosphorylation has been shown to induce processing of human p100^30,31^. Of note, human IKKβ induced phosphorylation and processing of Ap-NF-κB more efficiently than human IKKα (Supplementary Fig. 6). Furthermore, there is a single IKKα/β homolog in Aiptasia (and other basal animals) (Supplementary Fig. 4), suggesting that basal IKKs and their NF-κB substrates have properties that they share with both canonical and non-canonical signaling proteins of mammals. Furthermore, Ap-NF-κB has a glycine-rich region (GRR) between the RHD and the ANK repeat domain, and a similar GRR acts as a stop signal for the proteasome-mediated C-terminal processing of human p100^32^. However, it is important to note that there are no known RelB-like proteins (or any Rel-like protein) in Aiptasia^33^ nor any other basal organism, and we have been unable to identify a NIK-like protein in any basal organism.

Our results and others suggest that there has been co-evolution of the IKKs and their NF-κB/IκB substrates. For example, the nematode *C. elegans* has neither IKKs nor NF-κB/IκB proteins. The sponge *Amphimedon queenslandica* has an NF-κB protein without apparent C-terminal IKK phosphorylation sites (Supplementary Fig. 3), and *Amphimedon* lacks an IKKα/β ortholog (Supplementary Fig. 4). Moreover, the Relish protein of *Drosophila* lacks a GRR and C-terminal IKK phosphorylation sites, and the single *Drosophila* IKKα/β homolog does not appear to be involved in NF-κB processing or signaling^29^.

The truncated Ap-NF-κB protein (Δ427) enters the nucleus, binds DNA efficiently, and has intrinsic transactivation activity (Figs 1c, 2a,b). Thus, the Aiptasia NF-κB pathway appears to consist of a p100-like homodimer and an upstream IKK-like kinase, and activation of the Ap-IKK-like kinase presumably leads to phosphorylation of the Ap-NF-κB C terminus and proteolytic processing. Nevertheless, even though we can promote processing of Ap-NF-κB by overexpression of Ap-IKK in human cells in culture, the vast majority of Ap-NF-κB in anemones is in the processed form (Fig. 2f) and in the nucleus of cells (Fig. 3f) regardless of whether the anemones harbor *Symbiodinium* or have been rendered aposymbiotic by heat or menthol treatment. Whether Ap-NF-κB is always constitutively processed *in vivo* in anemones or there are factors or conditions that regulate its processing is not clear at this time. It is interesting to note that the majority of mouse NF-κB p100 is in its processed form when analyzed in extracts taken directly from liver, lymph nodes, and bone marrow^30^, but is primarily in its non-processed form when overexpressed in cells in culture^30^.

Our findings that laboratory-induced bleaching of Aiptasia is associated with increased expression and activity of NF-κB and that infection of naïve aposymbiotic *Aiptasia* larvae with *Symbiodinium* reduces NF-κB expression are consistent with recent findings showing that introduction of *Symbiodinium* into Aiptasia larvae resulted in decreased NF-κB mRNA five days later^34^. A simple interpretation of these collective results is that *Symbiodinium* down-regulates NF-κB expression in Aiptasia. We propose that NF-κB controls an immune pathway that must be suppressed for the establishment of symbiosis in Aiptasia. Consistent with this proposal, symbiotic Aiptasia (i.e., with reduced NF-κB) have a reduced capacity to respond to immune elicitors as compared to aposymbiotic (i.e., high NF-κB) anemones^35^. In addition, RNA-seq data comparing aposymbiotic and symbiotic adult Aiptasia show that the establishment of symbiosis is associated with down-regulation of an inflammation gene set^36^. In at least two symbiotic corals, *Orbicella faveolata* and *Acropora palmata*, increased levels of NF-κB transcripts have been found in bleached animals^10,25^.

It is important to note that the increase in Ap-NF-κB protein and activity that is seen with loss of symbiosis is not analogous to the rapid activation of NF-κB that occurs in many vertebrate and insect systems, i.e., wherein cytoplasmic NF-κB is rapidly freed from IκB inhibition to enter the nucleus. That is, the increases in Ap-NF-κB expression and activity following loss of symbiosis appear to be due to a corresponding increase in the total number of cells with nuclear Ap-NF-κB that arise following menthol or heat treatment. Thus, loss of symbiosis appears to result in increased transcription of Ap-NF-κB in a subset of gastrodermal cells only after a significant loss of symbiosis has occurred, and the resultant increase in expression of Ap-NF-κB protein is maintained for over two months in the absence of *Symbiodinium*. How *Symbiodinium* may suppress Ap-NF-κB mRNA and/or protein levels is not clear at this time.

NF-κB is involved in the control of innate immunity across a broad range of species^11^, and many pathogens and symbionts have mechanisms to modulate innate immunity, in some cases likely through effects on NF-κB, to ensure their survival within in the host. Indeed, NF-κB activity has been shown to be reduced by viral infection in humans, by an algal symbiont in salamanders, by bacterial symbionts in squid, and by commensal bacteria in the *Drosophila* gut microbiome^37–42^. Future studies will be aimed at determining the role of Ap-NF-κB in immunity and determining if NF-κB activity is increased by bleaching in other cnidarian dysbiosis settings, e.g., in corals, which are more deleteriously affected by loss of symbiosis.

## Methods

### Plasmid constructions and cell culture

Details about plasmids and plasmid constructions, as well as primers used for PCR amplification, EMSAs and qPCR, are included in Supplementary Tables 1 and 2. Plasmids were verified by restriction enzyme mapping and/or DNA sequence analysis.

DF-1 chicken fibroblasts and human 293 cells were grown in Dulbecco’s modified Eagle’s Medium (DMEM) (Invitrogen) supplemented with 10% fetal bovine serum (Biologos), 50 U/ml penicillin, and 50 μg/ml streptomycin. Transfection of cells with expression plasmids was performed using polyethylenimine (Polysciences, Inc.) as described previously^23,24^. Cells were seeded to be ~60% confluent on the day of transfection at which time cells were incubated with DNA/PEI at a ratio of 1:6 in serum-free media (300 µl for a 60 mm plate) for 15 min at room temperature. Following incubation, this mixture was added to cells with 3 ml DMEM/10% FBS. Twenty-four hours later, the medium was replaced with 3 ml of fresh DMEM/10% FBS. The following day, cells were harvested. If cells were used for immunofluorescence, they were passaged onto glass coverslips on the day prior to fixation. If cells were used for Western Blotting they were lysed by boiling for 10 min in 2x SDS sample buffer followed by a 10 min centrifugation to clarify the samples. If cells were used for EMSA they were lysed in AT lysis buffer supplemented with protease inhibitors (20 mM HEPES, pH 7.9, 150 mM NaCl, 1 mM EDTA, 1 mM EGTA, 20% wt/vol glycerol, 1% [wt/vol] Triton X-100, 20 mM NaF, 1 mM Na_4_P_2_O_7_·10H_2_O, 1 mM dithiothreitol, 1 mM Na_3_VO_4_, 1 mM phenylmethylsulfonyl fluoride, 1 μg/ml leupeptin, 1 μg/ml pepstatin A, 10 μg/ml aprotinin) followed by passing the sample five times through a 27.5 gauge syringe (as described previously^23,24^. Samples were centrifuged at 14,000 rpm and the supernatants were used for EMSAs, as described below.

### Anemone maintenance and preparation of anemone lysates

*Exaiptasia pallida* strain H2 (originally from Kaneohe Bay, Oahu, HI) animals, symbiotic with *Symbiodinium minutum* (clade B1), were maintained in 150 mls of artificial sea water (ASW) in glass dishes at 25 °C with light provided by Sylvania Gro-Lux (GRO/Aq/RP) fluorescent bulbs at approximately 20 µmol photons/m^2^/sec with a 12 h:12 h light:dark cycle. Menthol-induced loss of symbiosis was achieved by treatment of symbiotic anemones in a glass dish with 150 ml of 0.58 mM menthol in ASW (that had been aerated for 4 h prior to use). Anemones were incubated in the dark with the menthol/ASW solution with fresh menthol changes daily for three 24 h treatment periods^26^. After the last treatment, anemones were placed into fresh, aerated ASW and collected at either day 4, day 7, or day 14 (days numbered from beginning of treatment). Long-term aposymbiotic anemones were isolated by bleaching anemones in menthol and then storing the animals in the dark with regular three times per week artemia feedings for 73 days before assaying by the levels of NF-κB protein by Western blotting. Heat treatments were performed by placing symbiotic anemones into 150 ml of ASW in a glass bowl and the temperature was raised from 25 °C to 32 °C by increasing the temperature 1 °C every hour for 7 h. Then anemones were kept at 32 °C for 6 days (with daily water changes to maintain salinity levels at 32 ppt) to induce bleaching, after which they were collected for assaying. Anemones (average oral disc: 3 mm) were then lysed for Western blotting or EMSA, or were flash frozen for later use in qPCR. For the preparation of lysates for Western blotting, single anemones were homogenized in 20 µl of 2x SDS sample followed by boiling for 10 min and a 10-min centrifugation to remove cell debris. For extracts for EMSAs, Aiptasia were first homogenized in AT lysis buffer (as above), and samples were then centrifuged at ~19,000 × g, and the supernatants were used for EMSAs.

### Generation of anti-Ap-NF-κB antibody

A GST-Ap-NF-κB RHD fusion protein (containing amino 2–422 of Ap-NF-κB), encoded by plasmid pDEST15-Ap-NF-κB-RHD, was expressed in BL21 bacterial cells. The GST-Ap-NF-κB protein was then purified using glutathione affinity column chromatography. Seven mg of purified GST-Ap-NF-κB protein was sent to Thermo Fisher Pierce for custom antibody production in rabbits, and the resulting antiserum was further purified by passage through an anti-Ap-NF-κB column and an anti-GST column to obtain antibodies to only Ap-NF-κB.

### Western blotting

Western blotting was performed essentially as described previously^23^. Briefly, cell extracts were separated on a 7.5% SDS-polyacrylamide gel. Proteins were then transferred to nitrocellulose in transfer buffer (20 mM Tris, 150 mM glycine, 10% methanol) at 4 °C at 250 mA for ~1 h followed by 160 mA overnight. The membrane was blocked in TBST (10 mM Tris-HCl [pH 7.4], 150 mM NaCl, 0.1% v/v Tween 20) containing 5% powered milk (Carnation) for 1 h at room temperature. Filters were incubated at 4 °C with primary antiserum diluted in 5% milk TBST as follows: FLAG antiserum (1:1000, Cell Signaling Technology), Ap-NF-κB antiserum (1:10,000; see above) or human β-tubulin antiserum (1:1000, Cell Signaling Technology) that cross-reacts with Aiptasia β-tubulin. Membranes were washed three times for 10 min each in TBST and then incubated with anti-rabbit HRP secondary antiserum (Cell Signaling) at 1:2500 in TBST/5% milk for 1 h at room temperature with gentle shaking. Membranes were washed as above and reactive bands were detected using SuperSignal West Dura Extended Duration Substrate (Thermo Fisher Pierce) on Blue Basic Autoradiography Film (BioExpress). The identities of protein bands were assessed in relation to either known protein molecular weight standards or internal control proteins on the Western blots. Quantification of bands on Western blots was performed using ImageJ on scans of the original film images. For each sample, Ap-NF-κB protein expression was normalized to β-tubulin, and the average and standard error of each treatment were calculated across three biological replicates per treatment. Each treatment was compared to control, symbiotic anemones and a statistical analysis was performed with an unpaired, two-tailed t-test using the GraphPad QuickCalcs online software. Differences in protein expression were considered statistically significant if p < 0.05 and are denoted by (*) in figures. For representation of Western blots in figures, films were first scanned using uniform scanning settings with an Epson Perfection V500 scanner, and images were mocked up using Adobe Illustrator C56 software.

### Electrophoretic mobility shift assays

EMSAs were performed essentially as described^22^. A double-stranded κB-site oligonucleotide (GGGAATTCCC) was end labeled with T4 polynucleotide kinase (New England Biolabs) in 15 μl reaction with 1x T4 polynucleotide kinase buffer, 1 μg double-stranded probe, and 20 μCi [γ-32P]-dATP (Perkin Elmer) at 37° for 2 h. The reaction volume was then brought up to 100 μl with TE and excess label was removed by filtration through a Bio-Spin 6 Chromatography Column (Bio-Rad). For binding reactions using lysates from 293 cells, approximately 5–10 μg total protein and 100,000 cpm of the NF-κB-site probe were used. For Aiptasia lysates made in AT lysis buffer, approximately 50–60 μg of total protein and 200,000 cpm of the radiolabeled NF-κB-site probe were used. Reactions were carried out in either animal lysate binding buffer (10 mM Hepes pH 7.8, 50 mM KCl, 1 mM DTT, 1 mM EDTA, 4% w/v glycerol) or cell lysate binding buffer (10 mM Tris pH 7.4, 50 mM NaCl, 1 mM DTT, 1 mM EDTA, 4% w/v glycerol) for 30 min at 30 °C. Supershifts were performed by incubating the samples with 2 µl of antiserum for 2 h on ice after completion of the binding reaction. Samples were analyzed on 5% polyacrylamide gels, and bands were visualized on dried gels by autoradiography or phosphorimaging. Final images were prepared by uniform scanning and mocking up of images using Adobe Illustrator C56 software.

### Protein-binding microarray experiments and analysis

PBM experiments were carried out using custom NF-κB oligonucleotide arrays designed as part of this study (Agilent Technologies, AMADID 045485), based on a previously published “10-mer” κB microarray used to assay human and mouse NF-κB proteins^15^. DNA probe sequences synthesized on the custom-designed arrays are listed in Supplementary Data 1. Proteins used for PBM analysis were expressed in BL21 cells from Gateway pDEST15 GST-tagged vectors. BL21 cells were grown at 37 °C in LB containing chloramphenicol (50 µg/ml) and ampicillin (100 µg/ml), and GST protein expression was induced with 1 mM IPTG. Cells were mechanically lysed using a French press in the presence of protease inhibitors. GST-tagged proteins were purified with glutathione agarose (Thermo Fisher Scientific) and stored at −80 °C in 10% glycerol, before use. Probes on PBMs were made double-stranded by incubating the array with dNTP annealing mix at 85 °C for 10 min, 75 °C 10 min, 65 °C 10 min, then 60 °C for 90 min. The array was blocked at room temperature in filtered PBS with 2% milk for 1 h followed by washing in 0.1% PBS, Tween-20 (5 min) and 0.01% PBS, Triton-X (2 min). Arrays were incubated in the dark for 1 h at room temperature with ~300 nM protein and protein binding buffer (6 mM HEPES pH 7.8, 80 mM KCl, 0.5 mM EDTA, 0.5 mM EGTA, 6% glycerol, 35 ng/µl poly-dIdC, 1% milk,). Arrays were then washed with 0.05% PBS Tween-20 (3 min) and 0.01% PBS TX-100 (2 min). An A488-conjugated anti-GST (Invitrogen) antibody was used at 1:40 dilution in PBS containing 2% milk and incubated in the dark for 20 min at room temperature. Arrays were then washed as above followed by an additional wash in PBS (2 min) and then scanned on a GenePix 4400 A Scanner (Molecular Devices) and fluorescence was quantified with GenePix Pro 7.2 (Molecular Devices). PBM probe fluorescence values were spatially averaged and normalized using MicroArray LINEar Regression^43^ as described^44^. Replicate experiments were combined using quantile normalization of probe fluorescence values using the *normalize.quantiles* method in the ‘R’ statistical package (www.r-project.org). For each unique DNA binding sequence, median fluorescence values were determined over eight replicate probe measurements. Log median fluorescence values (i.e., log(F)) were transformed into a ‘z-score’ using the mean (μ) and variance (σ) of the log median fluorescence values for the 1195 random background DNA sequences: z = (log(F) −  μ)/σ).

### Indirect immunofluorescence

Indirect immunofluorescence was performed on transfected DF-1 cells as described previously^23^. Two days after transfection, cells were passaged onto glass coverslips in 35-mm dishes. The next day, coverslips were washed 3x with PBS, and then fixed in 100% methanol at −20 °C for 10 min. Fixed cells were blocked in PBS containing 3% calf serum for 1 h and then incubated with anti-FLAG antiserum (Cell Signaling) at a 1:50 dilution in PBS for 1 h at 37 C. Cells were washed as above and incubated with anti-rabbit secondary antiserum at 1:80 dilution for 1.5 h and then washed again. Coverslips were mounted onto slides using Vectashield, and samples were visualized by confocal microscopy.

### Luciferase reporter assays

Luciferase reporter assays were performed essentially as described previously^23^. Briefly, 293 cells were transfected with 1 µg of pcDNA-FLAG expression plasmid, 0.5 µg of a 3X NF-κB-site luciferase reporter plasmid, and 0.5 µg of plasmid pGK-βgal (for transfection normalization). Two days after transfection, lysates were prepared and luciferase activity was measured with the Luciferase Assay System (Promega) according to the manufacturer’s instructions. Values were normalized according to β-galactosidase activity in all assays.

### *In vitro* kinase assays

*In vitro* kinase assays were performed essentially as described previously^23^. Human 293 cells were transfected with pcDNA-FLAG-IKK constructs and the kinases were immunoprecipitated with anti-FLAG beads (Sigma). Immunoprecipitates were incubated with 4 μg of GST-tagged Ap-NF-κB C-terminal peptides (Supplementary Table 1) and 5 µCi [γ-^32^P]ATP (Perkin Elmer) in kinase reaction buffer (25 mM Tris-HCl, pH 7.5, 20 mM β-glycerophosphate, 10 mM NaF, 10 mM MgCl_2_, 2 mM DTT, 500 µM Na_3_VO_4_, 50 µM ATP) for 30 min at 30 °C. Samples were then electrophoresed on a 10% SDS-polyacrylamide gel, and ^32^P-labeled GST-Ap-NF-κB peptides were detected by phosphorimaging. In parallel, 4 μg samples of GST and the GST-Ap-NF-κB peptides were electrophoresed on a 10% SDS-polyacrylamide gel, and proteins were detected by staining with Coomassie blue (Bio-Rad).

### mRNA analysis

RNA was extracted using the TRIzol Reagent (Invitrogen) protocol using 500 µl of TRIzol per anemone. The final RNA samples were diluted in DEPC-treated H_2_O to a concentration of 50–100 ng/µl.

For qPCR, cDNA was prepared from 500 ng RNA for each sample. RNA was combined with 1.68 µl of 15.4 µM random primers (Promega) and nuclease-free water to 16 µl. Samples were incubated at 65 °C in a heating block for 5 min and then incubated on ice for 5 min. cDNA synthesis was initiated by the addition of 6 µl 2.5 mM dNTPs, 1 µl RNasin (Promega), 6 µl 5X M-MLV buffer (Promega), and 1 µl M-MLV reverse transcriptase (Promega), and samples were incubated at 37 °C for 1 h. cDNA samples were diluted 10-fold and used as the template for qPCR. 10 µl reactions were prepared (5 µl 2X-PowerUp SYBR Green Master Mix (Thermo Fisher Scientific), 1 µl each forward and reverse primer (at 1 µM), 2 µl cDNA (1:10), and 1 µl nuclease free water. All experiments included at least three biological replicates of each condition and each biological replicate was run in triplicate. No-template controls were included for each primer pair. Reactions were run in standard mode on a 7900-HT Real Time PCR System (Applied Biosystems) using the following thermocycling conditions: 2 min UDG activation at 50 °C, 2 min Dual-Lock DNA polymerase activation at 95 °C, and 40 cycles of denaturation and annealing/extension at 95 °C for 15 sec and 60 °C for 60 sec, respectively. Dissociation curves were generated by 15 sec incubation at 95 °C, 15 sec incubation at 60 °C, and then ramping from 60 °C to 95 °C at a 2% ramp rate.

mRNA expression data were analyzed following the Livak (∆∆Ct) method^45^. For each sample run in triplicate, the average Ct value for the target gene (Ap-NF-κB or *Symbiodinium* 28S RNA) and the average Ct value for the reference gene (L10) was calculated. The Ct value for the reference gene was then subtracted from the value for the target gene to obtain ∆Ct (i.e., Ct_NF-kB_−Ct_L10_). The average and standard deviation of ∆Ct for each set of biological replicates were calculated. The average ∆Ct for bleached samples was then subtracted from the average ∆Ct for the symbiotic samples to obtain the ∆∆Ct. The fold change in Ap-NF-κB mRNA or symbioint rRNA relative to the symbiotic samples was determined to be 2^−∆∆Ct^. Statistical analysis was performed with an unpaired two-tailed t-test using the GraphPad QuickCalcs online software. Differences in gene expression were considered statistically significant if p < 0.05.

### Staining of Aiptasia tissue sections

Anemones were relaxed in ASW with 10% w/v MgCl_2_ and fixed in 4% paraformaldehyde at 4 °C for 5 h. Anemones were dehydrated in 30% sucrose at 4 °C overnight. Samples were frozen in OCT using dry ice and ethanol and then stored at −20 °C before sectioning. Whole-body sections were cut on a cryostat at 10–20 µm, adhered to SuperFrost plus slides (Thermo Fisher Scientific), and incubated at room temperature for 30 min before staining. Slides were hydrated in PBS for 2 h and then blocked and permeabilized with PBS/0.3% Triton containing 5% goat serum and 1% BSA at room for 4–5 h. Slides were incubated overnight at room temperature with NF-κB antiserum or pre-immune serum at a 1:50,000 dilution in PBS containing 5% goat serum and 0.3% Triton. Slides were then washed three times for 10 min with PBS/0.05% Tween 20. Alexa Flour 488-conjugated goat anti-rabbit secondary antiserum (Invitrogen) was added at 1:500 with 5 µM Hoechst in PBS containing 5% goat serum and 0.3% Triton. Samples were incubated for 1.5 h at room temperature, and then slides were washed as above. Slides were fixed with prolong gold, and coverslips were applied. Slices in Fig. 3f (bottom) were imaged with a Zeiss LSM 700 laser scanning confocal microscope and the ZEN software package (Black Edition) using A488, DAPI, and mCherry. NF-κB puncta were quantified with ImageJ. Epifluorescent images (Fig. 3f, top) were taken on a Nikon Eclipse microscope.

### Anemone spawning, infection of larvae with *Symbiodinium*, and immunofluorescence staining

Aiptasia adults were spawned in the laboratory to generate larvae using a method similar to that described by Grawunder *et al*.^46^. Anemones from the clonal male line (CC7), clonal female line (PLF3), or wild-collected anemones from Florida were used for spawning. Larvae were reared in glass finger bowls in ASW until 3–4 days post-fertilization under a 25 µmol photons m^−2^ s^−1^ on a 12:12 h light:dark schedule at 27 °C. The larvae were then split into two groups, one was infected with *Symbiodinium* and the other served as an uninfected control. Prior to infection, axenic *Symbiodinium* (strain SSB01^47^) cells were rinsed three times by centrifugation at 3000 × g in ASW and were counted using a Guava Flow Cytometer (Millipore)^48^. Infections were performed at 50,000 cells/ml for 5–6 days in 50 ml of ASW. Control and infected larvae were fixed in 4% formaldehyde for 4 h at room temperature. Fixed larvae were permeabilized and blocked as described above for staining of anemone tissue sections. Larvae were then incubated overnight at room temperature with rabbit Ap-NF-κB (1:10,000) and goat anti-tubulin (Sigma; 1:200) primary antisera. Larvae were then washed three times in PBS, and were incubated with Alexa fluor-488-conjugated goat anti-rabbit secondary antiserum (Invitrogen; 1:500), Alexa fluor-649-conjugated mouse anti-goat secondary antiserum (Invitrogen; 1:200), and Hoechst (Sigma; 5 µM) followed by three washes in PBS. Larvae were then pipetted onto 10 µl of Prolong Gold on SuperFrost Plus slides and mounted with a coverslip. Larvae were imaged as above using A488, A649, DAPI, and mCherry channels. Corrected Total Cell Fluorescence (CTCF) of NF-κB was quantified using ImageJ. For each image, larvae were outlined using the circle tool and area, integrated density, and mean gray value were measured as well as the mean gray value for background fluorescence. CTCF was calculated by the formula (CTCF = Integrated Density − (Area × Mean gray value of background)). Five aposymbiotic and five symbiotic larvae from two trials were used. Statistical significance was determined using an unpaired, two-tailed T-test. ***p < 0.0001.

## Electronic supplementary material

Supplementary Information

Dataset 1

Dataset 2

Dataset 3

Dataset 4

Dataset 5
