# Supplementary material for: Transcription factor NF-κB is modulated by symbiotic status in a sea anemone model of cnidarian bleaching
Source: Sci Rep. 2017 Nov 22;7:16025. doi: 10.1038/s41598-017-16168-w (PMC5700166; doi:10.1038/s41598-017-16168-w)
Supplement: Supplementary file 1 — Supplementary Information [file 41598_2017_16168_MOESM1_ESM.pdf]

*Supplementary Information*

# Transcription factor NF-κB is modulated by symbiotic status in a sea anemone model of cnidarian bleaching

Katelyn M. Mansfield[1], Nicole M. Carter[1], Linda Nguyen[1], Phillip A. Cleves[2], Anar Alshanbayeva[1], Leah M. Williams[1], Camerron Crowder[3], Ashley R. Penvose[1], John R. Finnerty[1], Virginia M. Weis[2], Trevor W. Siggers[1] & Thomas D. Gilmore[1,*]

[1]*Department of Biology, Boston University, Boston, Massachusetts, 02215*

[2]*Department of Genetics, Stanford University School of Medicine, Stanford, California, 94305*

[3]*Department of Integrative Biology, Oregon State University, Corvallis, Oregon, 97331*

## Supplemental Methods

**Protein-binding microarray (PBM)-based clustering.** For all PBM experiments, DNA probe sequences were determined that were bound with a z-score >4.0, which was used to define a z-score profile (see Methods in main text). Pearson correlation values were determined for each pair of experiments (i.e., between each pair of z-score profiles). Hierarchical clustering of the correlation matrix was performed using the *heatmap* method in the R statistical package (www.r-project.org/).

**Phylogenetic analyses.** For comparative analyses of IKK-like kinases, we used predicted full-length protein sequences (MEME analysis[1]; Supplementary Data 3 and 4) or the amino acid region between MEME motifs 1-7 that that encodes the most conserved part of the serine/threonine kinase domain that is common to IKKα, IKKβ, IKKε and TBK proteins from *Homo*, *Ciona*, *Drosophila*, *Nematostella*, *Aiptasia*, *Amphimedon*, and *Trichoplax* (for maximum likelihood analysis; Supplementary Data 5). IKK-like proteins were also found in *Mnemiopsis*, *Pleurobrachia*, and *Capsaspora* (Supplementary Data 3). To find homologous IKK protein sequences, *Homo* IKKα, IKKβ, IKKε and TBK protein sequences were used as queries in tBLASTn searches on various, anciently diverged taxa. For confirmation of the search outputs, top hits from each taxa were reciprocally BLASTed to retrieve IKK proteins in *Homo*. We completed an alignment using Clustal Omega[2] to search for satisfactory motif recovery, motif alignment, and register. Phylogenetic trees were created with and without IKK-like proteins following observation of poor bootstrap support for branches containing ctenophore IKK-like proteins due to long-branch attraction. A maximum likelihood phylogenic tree was created using PAUP* and was bootstrapped 1000 times[3].

Based in part on our previously published MEME analysis[4], we compared NF-κB-like proteins across phyla for key structural elements, including GRRs and sites of IKK phosphorylation on NF-κB. GRRs were found by visually inspecting sequences just downstream of the C terminus of the RHD. Sites of phosphorylation by IKK were found by using known sites as identifiers for sites of NF-κB phosphorylation by IKKα to search for unknown sites in previously unmined NF-κB C-terminal protein sequences with Clustal Omega[2].

**Supplementary Tables**

**Supplementary Table 1. Plasmids used in this study.**

Expression Vectors for Use in Tissue Culture Cells

| *Plasmid Name* | *Plasmid Description* |
|---|---|
| pPacPL-FLAG-Relish | Encodes N-terminal FLAG-tagged Relish protein. From Neal Silverman (University of Massachusetts Medical School). |
| pMD5-Aq-NF-κB | Contains cDNA for *Amphimedon queenslandia* NF-κB. From Bernard Degnan (University of Queensland). |
| pUCIDT-Co-NF-κB | pUCIDT with *Capsaspora owczarzaki*-NF-κB codons 200-346 that were codon-optimized for expression in *E. coli*. Synthesized by Integrated DNA Technologies. |
| pUC57-Ap-NF-κB | pUC57 with a full-length Ap-NF-κB cDNA with a 5’ EcoRI restriction site and a 3’ BamHI restriction site. Synthesized by GenScript. |
| pUC57-Ap-IKK | pUC57-Simple with Ap-IKK cDNA codon-optimized for expression in human cells. Has a 5’ EcoRI site and 3’ XhoI site for excision. Synthesized by GenScript. |
| pcDNA-FLAG | pcDNA with a 5’ FLAG Tag[5]. |
| pcDNA-FLAG-Nv-NF-κB | Nv-NF-κB codons 3-440 subcloned into pcDNA-FLAG[5.]. |
| pcDNA-FLAG-Ap-NF-κB | EcoRI-BamHI fragment containing full-length Ap-NF-κB was excised from pUC57-Ap-NF-κB and subcloned into EcoRI-BamHI digested pcDNA-FLAG. |
| pcDNA-FLAG-Ap-NF-κB-SSS/AAA | EcoRI-Ap-NF-κB-1-2421-3X-Ser-Ala and Ap-NF-κB-2395-2547-3x-Ser-Ala-BamHI PCR fragments were used as a template for assembly PCR of an EcoRI-BamHI fragment containing full-length Ap-NF-κB-3x-Ser-Ala. Primers used for amplification were pcDNA-FLAG-Ap-NF-κB-F, Ap-NF-κB-3x-Ser-Ala-F, pcDNA-FLAG-Ap-NF-κB-R-Full, Ap-NF-κB-3x-Ser-Ala-R. |
| pcDNA-FLAG-Ap-NF-κBΔ427 | EcoRI-BamHI digested Ap-NF-κB-Δ427 PCR product containing codons 2-422 was subcloned into EcoRI-BamHI digested pcDNA-FLAG. Primers: pcDNA-FLAG-Ap-NF-κB-F and pcDNA-FLAG-Ap-NF-κB-R-427. PCR-amplified from pUC57-Ap-NF-κB. |
| pcDNA-FLAG-IKKα | Ref. 6 |
| pcDNA-FLAG-IKKβ | Ref. 6 |
| pcDNA-FLAG-Ap-IKK | EcoR-XhoI fragment containing Ap-IKK (codons 2-674) was excised from pUC57-Ap-IKK and subcloned into EcoRI-XhoI digested pcDNA-FLAG. |

| | |
|---|---|
| pcDNA-FLAG-Aq-NF-κB | BamHI-BamHI fragment containing codons 2-1193 of Aq-NF-κB was subcloned into BamHI-digested pcDNA-FLAG. Primers: pcDNA-FLAG-Aq-NF-κB-F and pcDNA-FLAG-Aq-NF-κB-R. PCR-amplified from pMD5-Aq-NF-κB. |
| pcDNA3.1-Ap-NF-κB | BamHI-EcoRI fragment containing Ap-NF-κB codons 1-849 was subcloned into BamHI-EcoRI digested pcDNA3.1(+). Primers: pcDNA-ATG-Ap-NF-κB-F and pcDNA-Ap-NF-κB-R. PCR-amplified from pcDNA-FLAG-Ap-NF-κB. |
| pcDNA3.1-Ap-NF-κB-SSS/AAA | BamHI-EcoRI Ap-NF-κB-SSS/AAA fragment was subcloned into BamHI-EcoRI digested pcDNA3.1(+). Primers: pcDNA-ATG-Ap-NF-κB-F and pcDNA-Ap-NF-κB-R were used to PCR-amplify the fragment from pcDNA-FLAG-Ap-NF-κB-SSS/AAA. |
| HA-NIK | From Shao-Cong Sun (MD Anderson). |
| HA-IKKβ-SS-EE | Ref. 6 |

Bacterial Expression Vectors

| *Plasmid Name* | *Plasmid Description* |
|---|---|
| pGEX-KG | Expression plasmid containing a 5’ GST tag. |
| pGEX-KG-Ap-NF-κB-C-term | EcoRI-HinDIIII fragment containing Ap-NF-κB codons 782-812 was subcloned into EcoRI-HinDIII digested pGEX-KG. Primers- pGEX-KG-Ap-C-term-F and pGEX-KG-Ap-C-term-R were used to PCR-amplify the fragment from pcDNA-FLAG-Ap-NF-κB. |
| pGEX-KG-Ap-NF-κB-C-term-SSS/AAA | EcoRI-HinDIII fragment containing Ap-NF-κB-SSS/AAA codons 782-812 was subcloned into EcoRI-HindIII digested pGEX-KG. Primers- pGEX-KG-Ap-C-term-F and pGEX-KG-Ap-C-term-R. PCR-amplified from pcDNA-FLAG-Ap-NF-κB-SSS/AAA. |
| pDEST15 | Gateway destination expression plasmid containing a 5’ GST tag. |
| pDEST15-Ap-NF-κB-RHD | PCR amplified from pUC57-Ap-NF-κB using Gateway primers to insert codons 2-422 into pDEST-15. Primers: Ap-Gateway-F and Ap-Gateway-R. |
| pDEST15-Aq-NF-κB | PCR amplified from pMD5-Aq-NF-κB using Gateway primers to insert codons 2-389 into pDEST15. Primers: Aq-Gateway-F and Aq-Gateway-R. |
| pDEST15-Co-NF-κB | PCR amplified from pUCIDT-Co-NF-κB using Gateway primers to insert codons 200-546 into pDEST15. Primers Co-Gateway-F and Co-Gateway-R. |
| pDEST15-Relish | PCR amplified from pPacPL-FLAG-Relish using |

| | |
|---|---|
| | Gateway primers to insert codons 265-649 into pDEST15. Primers: Relish-Gateway-F and Relish-Gateway-R. |
| pDEST15-Hu-p50 | Ref. 7 |
| pDEST15-Nv-NF-κB-S | PCR amplified from pcDNA3.1(-)-Nv-NF-κB-Ser67 using Gateway primers to insert codons 2-411 into pDEST15. Primers Nv-NF-κB-F and Nv-NF-κB-R. |
| pDEST15-Nv-NF-κB-C | PCR amplified from pcDNA3.1(-)-Nv-NF-κB-Cys67 using Gateway primers to insert codons 2-411 into pDEST15. Primers Nv-NF-κB-F and Nv-NF-κB-R. |

**Supplementary Table 2. Primers used in this study.**

Primers for Subcloning (restriction enzyme sites used for subcloning are underlined)

| *Plasmid created* | *Primers* |
|---|---|
| pcDNA-FLAG-Aq-NF-κB-F | 5'- CGAAGGATCCCTGCTTTTAATGGTATTGA TCCC -3' |
| pcDNA- FLAG -Aq-NF-κB-R | 5'- CGAGGGATCCCTAAACTTGACTAGAAGG -3' |
| pcDNA- FLAG -Ap-NF-κB – F | 5'- CGGACGAATTCAACACATTCAGAACAGC AAGTC -3' |
| pcDNA- FLAG -Ap-NF-κB-R-427 | 5'- CAAGGATCCCTAATCAAACAGGAAACCA CTGCC -3' |
| pcDNA- FLAG -Ap-NF-κB-R-Full | 5'- CGAGGATCCTCAGTTCGTTCTTCCCATT GG -3' |
| Ap-NF-κB-3x-Ser-Ala-F | 5'- GACGCAGGCTTTGGGGCCCAGGCTGCA -3' |
| Ap-NF-κB-3x-Ser-Ala-R | 5'- TGCAGCCTGGGCCCCAAAGCCTGCGTC -3' |
| pcDNA-ATG-Ap-NF-κB-F | 5'- CGCGGATCCATGACACATTCAGAACAGC AA -3' |
| pcDNA-Ap-NF-κB-R | 5'- GCGCGGAATTCTCAGTTCGTTCTTCCCAT -3' |
| pGEX-KG-Ap-NF-κB-C-term-F | 5'- GCGCGCGAATTCTAAACTATGGCGACT AT -3' |
| pGEX-KG-Ap-NF-κB-C-term-R | 5'- GCGCGAAGCTTCTAATCATTGTCTCTTTC TGA -3' |
| Ap-Gateway-F | 5'-GGGGACAAGTTTGTACAAAAAAGCACGC TTCACACATTCAGAACAGCAA -3' |
| Ap-Gateway-R | 5'- GGGGACCACTTTGTACAAGAAAGCTGGG TCCTAATCAAACAGGAAACCACTG -3' |
| Aq-Gateway-F | 5'- GGGGACAAGTTTGTACAAAGCAGGCTTC GCTTTTAATGGTATTGATCCC -3' |
| Aq-Gateway-R | 5'- GGGGACCACTTTGTACAAGAAAGCTGGG TCCTAACCCCCTCCACCGGGACCTTC -3' |
| Aq-Gateway-F | 5'- GGGGACAAGTTTGTACAAAAAAGCAGGC TTCGGCGTTAGTTTCGGCGTTGCT -3' |

| | |
|---|---|
| Relish-Gateway-R | 5'- GGGGACCACTTTGTACAAGAAAGCTGGG TCCTAATTAGAGCTTTCTGTTCCCGAA -3' |
| Co-Gateway-F | 5'- GGGGACAAGTTTGTACAAAAAAGCAGGC TTCTCCCCATCCGTTTCAACC -3' |
| Co-Gateway-R | 5'- GGGGACCACTTTGTACAAGAAAGCTGGG TCCTAACCGTCGCTGCCGTCGAAG -3' |
| Nv-NF-κB-F | 5'- GGGGACAAGTTTGTACAAAAAAGCAGGC TTCGCACAGTCTGAACAGCAAG -3' |
| Nv-NF-κB-R | 5'- GGGGACCACTTTGTACAAGAAAGCTGGG TCTAACCTGTAGCTCCAGATGAG -3' |

**Primers for EMSA**

| | |
|---|---|
| NF-κB-Consensus | 5'- TCGAGAGGTCGGGGAATTCCCCCCCCG -3'<br>5'- TCGACGGGGGGGGAATTCCCCGACCTC -3' |

**Primers for qPCR**

| | |
|---|---|
| Ap-NF-κB | 5'- CGACCCCACCAGAATCTGAAAG -3'<br>5'- ACGAATCATTGTCTCTTTCTGCAG -3' |
| *Symbiodinium* Clade B 28S[8] | 5'- CCTCTTGGACCTTCCACAAC -3'<br>5'- GCATGCAGCAACACTGCTC -3' |
| L10[8] | 5'- ACGTTTCTGCCGTGGTGTCCC -3'<br>5'- CGGGCAGCTTCAAGGGCTTCA -3' |

**Supplementary Figures**

**Supplementary Figure 1**

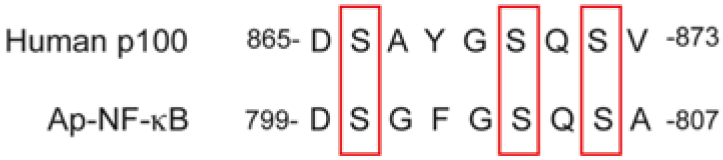


**Supplementary Figure 1. Alignment of the IKK phosphorylation sites of human p100 and Ap-NF-κB.** Human p100 serine residues that are phosphorylated by IKKα are conserved in Ap-NF-κB (red boxes).

**Supplementary Figure 2**

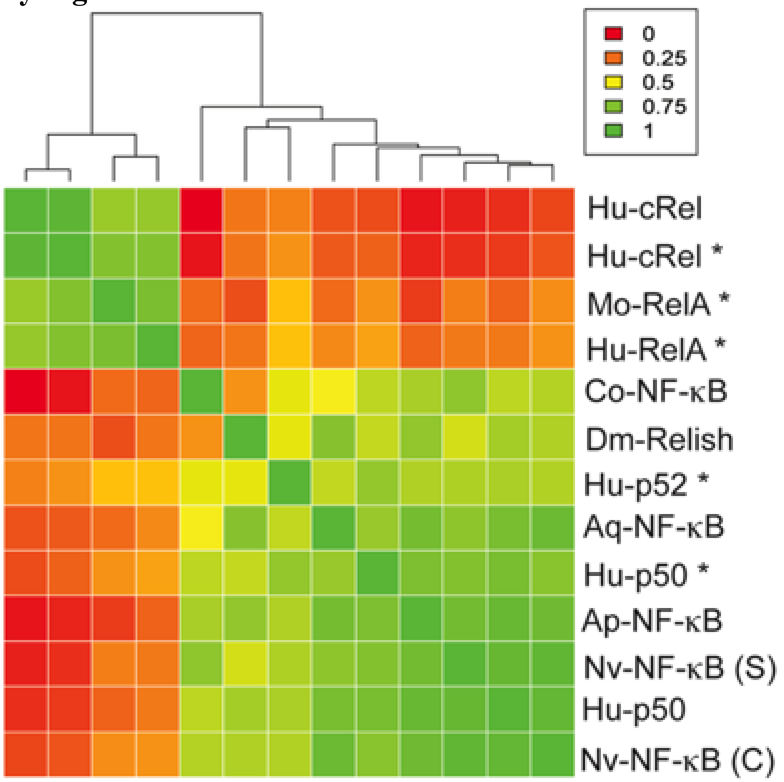


**Supplementary Figure 2**. **Comparison of DNA-binding site preferences of NF-κB Orthologs**. Pairwise comparisons of the DNA-binding specificity of bacterially expressed NF-κB family proteins from multiple species. Pairwise binding similarity was assessed by Pearson correlation of PBM-determined z-score values to 2592 NF-κB binding sites. Hierarchical clustering was carried out on the comparison matrix as described previously[7]. * indicates previously published PBM binding data[7], and in some cases (e.g., Hu-cRel and Hu-p50 were repeated in our lab to verify the reproducibility of our PBM analyses. (Abbreviations: Ap, *Aiptasia pallida*; Aq, *Amphimedon queenslandica*; Co, *Capsaspora owczarzaki*; Dm, *Drosophila melanogaster*; Mo, mouse; Hu, human; Nv, *Nematostella vectensis*. C and S indicate the polymorphic NF-κB proteins from *Nematostella*[5,9,10].

## Supplementary Figure 3

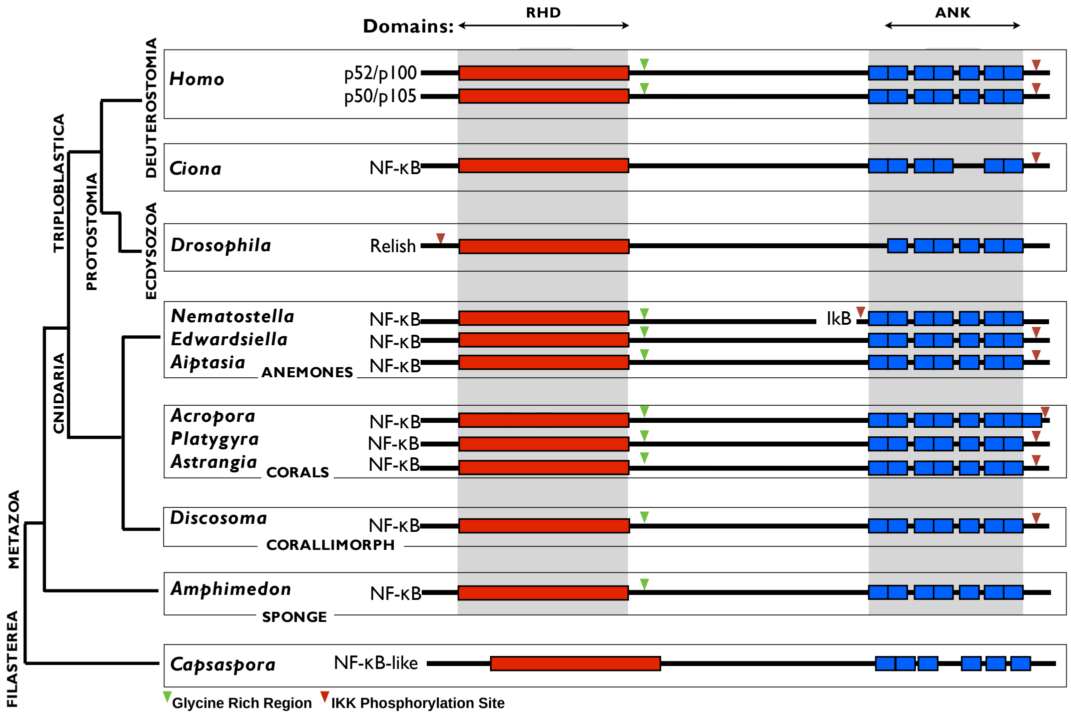


**Supplementary Figure 3. General structures of NF-κB proteins.** Conserved motifs in the NF-κB protein were identified by MEME analysis. Motifs within the Rel Homology domain (RHD) and Ankyrin repeats (ANK) were encapsulated into single motifs and boxed (grey shading). Clustal Omega was used to align the protein sequences, and the relevant regions were then visually inspected to identify glycine-rich regions (green inverted triangles) and predicted IKK phosphorylation sites (red inverted triangles) were inferred by sequence homology to known phosphorylation sites in human NF-κB proteins.

**Supplementary Figure 4**

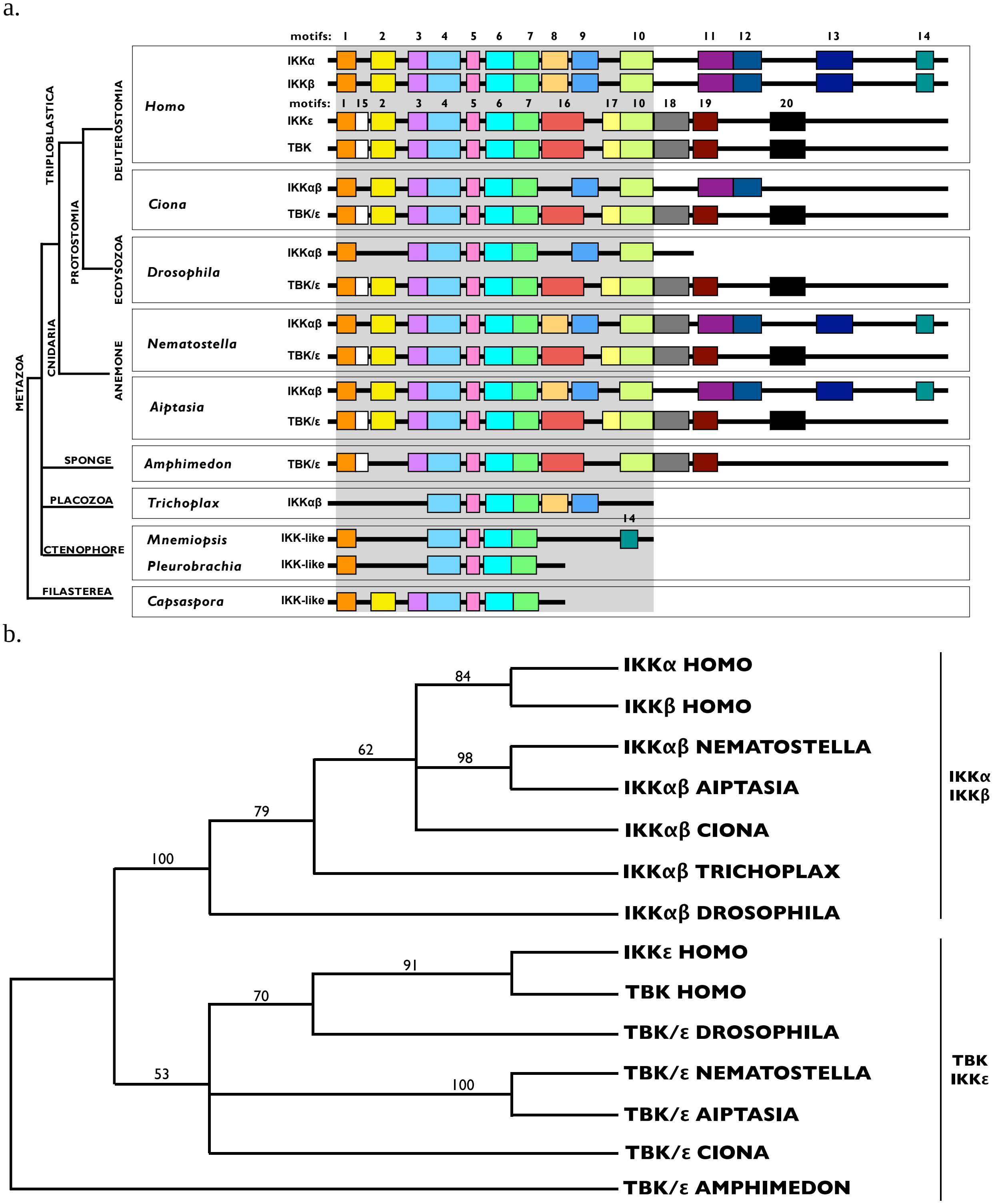


**Supplementary Figure 4. MEME analysis and phylogenetic comparison of IKK-like proteins.** (**a**) MEME analysis of IKK proteins from different taxa. Colored boxes indicate conserved protein motifs among the indicated IKK proteins. The shaded region indicates the serine/threonine kinase domain. IKKα/β and TBK/IKKε single orthologs are named as such

because they had extensive shared motifs with their vertebrate counterparts, had at least 33% similarity to the human counterparts by reciprocal BLAST analysis, and were in the same clades by phylogenetic analysis. (**b**) Phylogenetic analysis of IKK evolution using maximum likelihood tree that was bootstrapped 1000 times. The phylogeny was rooted with the TBK/IKKε of *Amphimedon* and branches indicate bootstrap support values. The IKKα/β and TBK/ε clades are clearly distinguished and mostly recapitulate evolution with Homo and sea anemone proteins sequences clustering. Proteins were named according to at least 33% sequence similarity for reciprocal BLASTs against the human genome using tBLASTn on NCBI.

**Supplementary Figure 5**

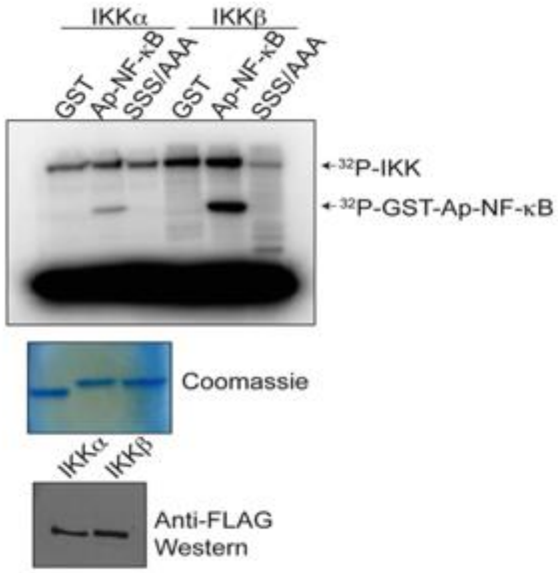


**Supplementary Figure 5. Human IKKα and IKKβ can phosphorylate Ap-NF-κB but not Ap-NF-κB-SSS/AAA *in vitro*.** As in Fig. 2c, GST-tagged peptides were incubated with FLAG-tagged IKKα or IKKβ in kinase reaction buffer (25 mM Tris HCl, pH 7.5, 20 mM β-glycerophosphate, 10 mM NaF, 10 mM $MgCl_2$, 2 mM DTT, 500 µM $Na_3VO_4$, 50 µM ATP) in the presence of gamma-[$^{32}$P]-ATP (which is the large band shown at the bottom of the gel). Samples were then electrophoresed on a 12.5% SDS polyacrylamide gel. The gel was dried and radioactivity was detected by phosphorimaging. The middle panel shows the GST peptides used in the assay (as in Fig. 2c). The bottom panel shows the relevant portion of an anti-FLAG Western blot of the kinases expressed in 293 cells that were used in the kinase assay.

**Supplementary Figure 6**

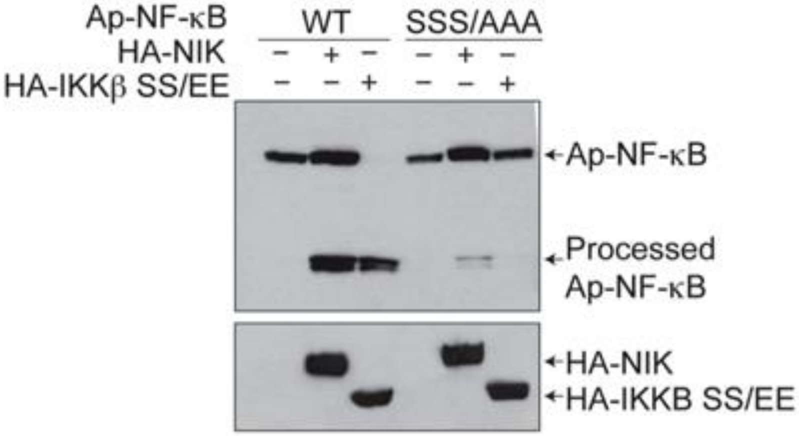


**Supplementary Figure 6. Human NIK and constitutively active IKKβ can induce processing of Ap-NF-κB but not the Ap-NF-κB-SSS/AAA mutant.** Plasmids were co-transfected into 293 cells and lysates were analyzed by Western blotting for Ap-NF-κB. The blot was stripped and reprobed for the HA-tagged kinases. The relevant portions of the images are shown and the relevant immunoreactive bands are shown.

**Supplementary Figure 7**

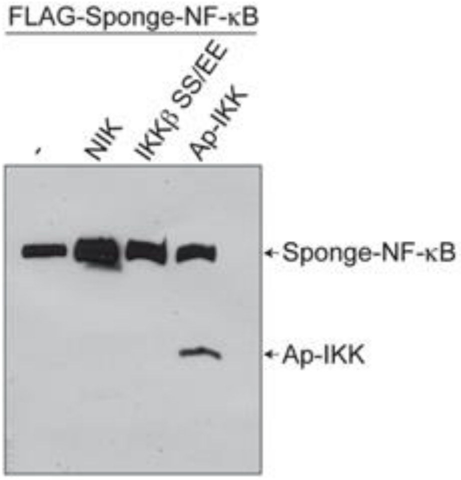


**Supplementary Figure 7. Sponge NF-κB is not processed by human NIK, constitutively active human IKKβ SS/EE, or Ap-IKK.** Plasmids encoding FLAG-tagged sponge (Aq) NF-κB and the indicated kinases were co-transfected into 293 cells and lysates were analyzed by anti-FLAG Western blotting. NIK and IKKβ SS/EE are HA-tagged constructs and Ap-IKK is FLAG-tagged.

**Supplementary Figure 8**

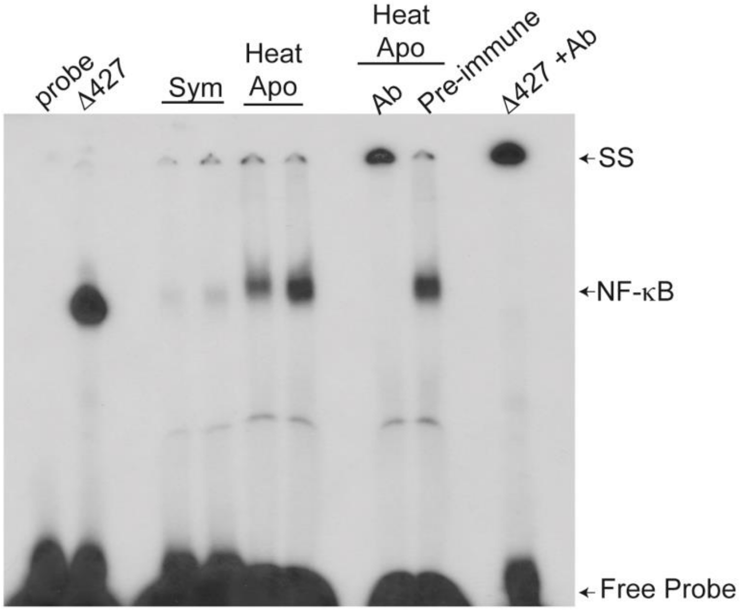


**Supplementary Figure 8. *Aiptasia* made aposymbiotic by heat stress have increased NF-κB DNA-binding activity as compared to symbiotic *Aiptasia*.** Symbiotic anemones were heat shocked (at 32$^{o}$C) for 6 days. Animal lysates were made and used in an EMSA as described in Methods and for Fig. 3e. Two animals were used for each condition. The position of the Ap-NF-κB-DNA complex was determined by co-migration with a complex generated from 293 cells transfected with a pcDNA-FLAG-Ap-NF-κBΔ427 expression plasmid. Where indicated, supershifts (SS) were performed with Ap-NF-κB antiserum (Ab) or pre-immune serum. The free-probe is shown at the bottom of the image.

**Supplementary Data Files**

Supplementary Data 1. DNA probes used for PBM analysis
Supplementary Data 2. MEME analysis Input
Supplementary Data 3. MEME analysis Output
Supplementary Data 4. Truncated IKK sequences used for phylogenetic analysis
Supplementary Data 5. Assembled *Aiptasia* IKK sequence used for *in vitro* and *in vivo* assays. Shown are the *Aiptasia* human codon-optimized IKK cDNA sequence (black), the native cDNA sequence (red), and the predicted 719 amino acid sequence (blue). The truncated IKK sequence used in kinase assays is designated by a black bar after amino acid 651.

**Supplementary References**


1. Bailey, T.L., Williams, N., Misleh, C. & Li. W.W. MEME: discovering and analyzing DNA and protein sequence motifs. *Nucleic Acids Res.* **349,** W369-W373 (2006).
2. Sievers, F *et al.* Fast, scalable generation of high-quality protein multiple sequence alignments using Clustal Omega. *Mol. Syst. Biol.* **7,** 539 (2011).
3.  Swofford, D.L. PAUP*. Phylogenetic analysis using parsimony (*and other methods). Sunderland, Massachusetts, Sinauer Associates (2002).
4. Finnerty, J.R. & Gilmore, T.D. Methods for analyzing the evolutionary relationship of NF-κB proteins using free, web-driver bioinformatics and phylogenetic tools. *Meth. Mol. Biol.* **1280**, 631-646 (2015).
5. Wolenski, F.S. *et al.* Characterization of the core elements of the NF-κB signaling pathway of the sea anemone *Nematostella vectensis*. *Mol. Cell. Biol.* **31,** 1076-1087 (2011).
6. Starczynowski, D.T. *et al.* Mutation of an IKK phosphorylation site within the transactivation domain of REL in two patients with B-cell lymphoma enhances REL's in vitro transforming activity. *Oncogene.* **26**, 2685-2694 (2007).
7. Siggers, T. *et al.* Principles of dimer-specific gene regulation revealed by a comprehensive characterization of NF-κB family DNA binding. *Nat. Immunol.* **13**, 95-102 (2011).
8. Poole, A.Z., Kitchen, S.A. & Weis, V.M. The role of complement in the cnidarian-dinoflagellate symbiosis and immune challenge in the sea anemone *Aiptasia pallida*. *Front. Microbiol.* **7**, 519 (2016).
9. Ryzhakov, G. *et al.* Cross-species analysis reveals evolving and conserved features of the nuclear factor κB (NF-κB) proteins. *J. Biol. Chem.* **288**, 11546-11554 (2013).
10. Sullivan, J.C. *et al.* Two alleles of NF-κB in the sea anemone *Nematostella vectensis* are widely dispersed in nature and encode proteins with distinct activities. *PLoS. One.* **4**, e7311 (2009).
