# Supplementary material for: Transcription factor NF-κB is modulated by symbiotic status in a sea anemone model of cnidarian bleaching
Source: Sci Rep. 2017 Nov 22;7:16025. doi: 10.1038/s41598-017-16168-w (PMC5700166; doi:10.1038/s41598-017-16168-w)
Supplement: Supplementary file 6 — Dataset 5 [file 41598_2017_16168_MOESM6_ESM.doc]

Codon optimized Native Amino Acid

ATGCAGGTAAGCGGACATGATGATTGGATAGAAGAACGGTGCCTCGGAGCGGGCGGTTTCGGAACGGTAACTTTG

--------T--------------C------------A-A---T-G—-G—-T—-T—-C—-T—-T—-A-----A--A

1 M Q V S G H D D W I E E R C L G A G G F G T V T L

TGGAAACACAAGTACAATCACGAATACCTGGCTATAAAAAAGTGTAGATTGGACCTCAGCCCGGCCAATCGCCAA

--------T--------------G---T----A--------A---C-TC-----T-G-----A—-A—-C—-T---

26 W K H K Y N H E Y L A I K K C R L D L S P A N R Q

CGATGGCACCAGGAAGTGGAGATCCTCAAGAAGCTCGATCACGCCAACATAGTCAAGGCGAAAGACGTACCGGCC

A----------A—-G-----A—-AT-G-----A-—T--------A-----T—-A—-A—-C-----T—-G—-A—-G

51 R W H Q E V E I L K K L D H A N I V K A K D V P A

ATACTCGACGTTAGTGGCGGAGAGATTCCCCTTCTGGCTATGGAGTACTGTGAAGGAGGTGATCTTAGGAGGATA

--T-----T—-C-----A--------C—-G—-G-----A--------T--------G--G--C--A--------C

76 I L D V S G G E I P L L A M E Y C E G G D L R R I

CTTAATACACCCGAGAATATCAGAGGACTCAAAGAATCAACAGTAATAGAAGTCACGGCCGACGTTGCTCACGCA

T-G-----T--T--A------C----GT-A--G--G--G--T--G--T-----G--T-----T--C-----T--C

101 L N T P E N I R G L K E S T V I E V T A D V A H A

ATCGAGTTCCTGCACAGTAAGAGGATAATCCATAGAGACCTCAAGCCGGAGAACATAGTCATTCAACACACAGAC

--T-----T--T---TCA---C-T-----T--CC-T---T=G-----T--A-----T--T-----G-----T--T

126 I E F L H S K R I I H R D L K P E N I V I Q H T D

GGGAAAGACATCTACAAGCTGATCGACCTTGGATATGCCAAACAGTTGGATCAATACTCCATTGCTACGACGTTC

--C--G--T---------T----T--T-----------T------C-C---------AG------A-----T--T

151 G K D I Y K L I D L G Y A K Q L D Q Y S I A T T F

GTGGGGACTCTTAGATACCTCGCGCCGGAACTCTTGGCCGGAAATGGAAGCTATACAAAAACCGTGGACTACTGG

--T--T---T-G------T-G--T--T---T-A-----T--G--------T--C--T-----T--T---------

176 V G T L R Y L A P E L L A G N G S Y T K T V D Y W

AGTCTGGGGACCGTTCTCTTTGAATGTATCACTGGAATCAGGCCATTTCCGGACCTGAGCCCGGTCAACTGGCAT

-----T-----A--G--G-----G--C--T-----G--AC-T-----C--A-----CTCT--T--G--T------

201 S L G T V L F E C I T G I R P F P D L S P V N W H

CGCGAGATTGGCCAGAAGTCCCCTAGACACATCCATGCATTCTACAATGCCTCCGACGAGATCACGTTTTCAGAA

A-A--A-----T--A-----A--A-----T--T--C--T--T--------A--T--T--A--A--C-----T---

226 R E I G Q K S P R H I H A F Y N A S D E I T F S E

GTCTTTCCCGAGCTTAACACTCTTTCTCGATGTTTCCAGGAAAAGTACGTAAACCTGTTGAGACTGTTGTTGCTC

--T--C--A--A--C-----------CA----C--T--A--G--A--------T--TC--C--T--C-----T-A

251 V F P E L N T L S R C F Q E K Y V N L L R L L L L

TGGGACCCCGTTAAACGCGGTGGTCAGGTACACGAGGACGGCTCACGGCAGTGCTATAAATTGTTGCATAAGATA

-----T--T------A-G-----C--A--G--T--A--T-----T-----A-----------A--A-----A---

276 W D P V K R G G Q V H E D G S R Q C Y K L L H K I

CTGGATTCTAAGGTTGTCCACATTTTTTGTGCTTTTACATCAACCCTTCTCACCTTTGAAGTAACTCAAACAGAC

T-------A-----G--T--T--A--------G--C--CAGT--TT-A--A--A--------T--A--G------

301 L D S K V V H I F C A F T S T L L T F E V T Q T D

CGACGCGAGGACATAAACGCTCGCATTTATGAGGAGACTGGTATTGCCCTTGACGACCAAGTAATAGTTTCCCCA

A--A-A--A--T-----T---A-G--------A--A-----A--C--TT-G--T-----G--C--T-----T--T

326 R R E D I N A R I Y E E T G I A L D D Q V I V S P

GCCGGGCAGGAAATCGGAGCAGACAGCCCAATGCTTGAGTACATTCATAAAAATAACGGCACTCCGTCCACCTTG

--T--A--------T--T--T--TTCA--C---T-G--A--T-----------------A--A-----G--GC-A

351 A G Q E I G A D S P M L E Y I H K N N G T P S T L

TATCTCTTTAGTAAGAGCAACATACCTGCATCACCTAGGCCACTGTTCACACTCCCCAGCACCTTGCAGTCAGTA

---T-A---TCC-----T--------A--T-----AC-T--T--C-----C--T--TTCT--A--A--A-----T

376 Y L F S K S N I P A S P R P L F T L P S T L Q S V

GTAACCGAGTCCAAGACACTCCTTCCGTACCTGGAGCAGAAACGGATCCACGCGGAGGCGCTCTCATTCTGTTAT

--T--A--A--G--A--T-----A--T--T--T-----A--GA-A--T--T--C-----A---AGT--T-----T

401 V T E S K T L L P Y L E Q K R I H A E A L S F C Y

AAGCAGATGAAAAATTACAAGTATCTGATTCAGGCGCACGGCACACTCTTGAAGTACACCCTCAAGTTGCACAGT

-----C--------C-----A-----------------T------T-AC-A--A--T--TT-G--AC-A---TCC

426 K Q M K N Y K Y L I Q A H G T L L K Y T L K L H S

CGATTGAGTCAATTGAGAACTAACCTTACCAGTGATTGCATTCGACTGGAGGAGCGGATTACATTTTGTAGAGAG

A----ATCA--GC-T--G--A---T-G--TTC------T--A--T--T-----A--A-----G-----C------

451 R L S Q L R T N L T S D C I R L E E R I T F C R E

TCACTCCATACGGACATAGAACATTTCTCTGATGTGGCCGTTGACCTCCACGCAAATGACATGATTCTTCGGAGT

AGC--T--C--G--T--T-----C--------C--C--A--A---T-G--T--------------C--C--TTCA

476 S L H T D I E H F S D V A V D L H A N D M I L R S

TGGAGAGAGGGTGAGAGAAATTGGATTCATTTTGATAAATCTGGCGTGATGGAAGCGGAGAAACTGGCTATTAGC

-----G--A--C--A-----------------C--C--G-----T--C--------A--A--G--C--C--CTCA

501 W R E G E R N W I H F D K S G V M E A E K L A I S

GCCCAGACGCAAGTACTGGAACTCCAGAAGATACCATTTAATCAGTCACAGCCAGTATCCAACCCGATGGAAGAG

-----A--C-----C--T---T-A-----A--T--G--------A-----A--C--G--T--T--A--------A

526 A Q T Q V L E L Q K I P F N Q S Q P V S N P M E E

ATTTATAACTCCGCGGTAATTGTATATGAGGGCTTTAAGCGCGCAAACTTGACATCCTACGATCAGAAGATGGCA

--A--------T--A--T-----T--------------AA-A-----T--A---AGC-----C------------

551 I Y N S A V I V Y E G F K R A N L T S Y D Q K M A

GATTGTTCAAAAATTGCCGAAGTAGTCTTGAAATGCCTGACCAAGAGGGAAAAACTCAACAAAGAGATGTTCACC

--C--CAGT-----A--T-----T--T---------T-T--A-----A-----GT-A-----G--A--------T

576 D C S K I A E V V L K C L T K R E K L N K E M F T

CATCTCCGACGCATTCTGCAGTGTTCTACAGAGCTTCAAAACGTGGTGCCGAAAGTGATAGAAAAAACAAGACAA

---T-AA----A-----T--A-----A-----G--A-----T--C--A--T-----T--T--G-----TC-G---

601 H L R R I L Q C S T E L Q N V V P K V I E K T R Q

ATATCCCATCACAAAGACCATATTCTTCAAATACAAAAGCAACGGCAGGAAGATGTTTGGTGCCTGATTTCCAAC

--T--T--------G-----C--A--G--G--C--G--A---A-A--A-----C--------T--C--A-----T

626 I S H H K D H I L Q I Q K Q R Q E D V W C L I S N

AAA|

--GCAAGACAGTGTTGACTCAGGAGTTAATTATCCCACGTTAGATTTAACATTATCAAGTTACAAAATGGACTCT

651 K Q D S V D S G V N Y P T L D L T L S S Y K M D S

TCAGAACTCACGAAACAGAGTTTGCAAAATATCGAAAGATTGTCTGAGGTCGTTCAGTCCGTGAAAGTGGAGTAC

676 S E L T K Q S L Q N I E R L S E V V Q S V K V E Y

GGTAACGTAGCGCCAGAGAGTTTAGATTGGAGTTTCCTAGAAGAGGACACTGAAGTCTGA

701 G N V A P E S L D W S F L E E D T E V *
